# What Makes a Bacterial Species Pathogenic?:Comparative Genomic Analysis of the Genus *Leptospira*

**DOI:** 10.1371/journal.pntd.0004403

**Published:** 2016-02-18

**Authors:** Derrick E. Fouts, Michael A. Matthias, Haritha Adhikarla, Ben Adler, Luciane Amorim-Santos, Douglas E. Berg, Dieter Bulach, Alejandro Buschiazzo, Yung-Fu Chang, Renee L. Galloway, David A. Haake, Daniel H. Haft, Rudy Hartskeerl, Albert I. Ko, Paul N. Levett, James Matsunaga, Ariel E. Mechaly, Jonathan M. Monk, Ana L. T. Nascimento, Karen E. Nelson, Bernhard Palsson, Sharon J. Peacock, Mathieu Picardeau, Jessica N. Ricaldi, Janjira Thaipandungpanit, Elsio A. Wunder, X. Frank Yang, Jun-Jie Zhang, Joseph M. Vinetz

**Affiliations:** 1 J. Craig Venter Institute, Rockville, Maryland, United States of America; 2 Division of Infectious Diseases, Department of Medicine, University of California San Diego School of Medicine, La Jolla, California, United States of America; 3 Department of Epidemiology of Microbial Diseases, Yale School of Public Health, New Haven, Connecticut, United States of America; 4 Australian Research Council Centre of Excellence in Structural and Functional Microbial Genomics, Department of Microbiology, Monash University, Clayton, Australia; 5 Centro de Pesquisas Gonçalo Moniz, Fundação Oswaldo Cruz/MS, Salvador, Bahia, Brazil; 6 Victorian Bioinformatics Consortium, Monash University, Clayton, Victoria, Australia; 7 Institut Pasteur de Montevideo, Laboratory of Molecular and Structural Microbiology, Montevideo, Uruguay; 8 Institut Pasteur, Department of Structural Biology and Chemistry, Paris, France; 9 Department of Population Medicine & Diagnostic Sciences, College of Veterinary Medicine, Cornell University, Ithaca, New York, United States of America; 10 Centers for Disease Control and Prevention (DHHS, CDC, OID, NCEZID, DHCPP, BSPB), Atlanta, Georgia, United States of America; 11 VA Greater Los Angeles Healthcare System, Los Angeles, California, United States of America; 12 David Geffen School of Medicine at UCLA, Los Angeles, California, United States of America; 13 WHO/FAO/OIE and National Collaborating Centre for Reference and Research on Leptospirosis, KIT Biomedical Research, Royal Tropical Institute (KIT), Amsterdam, The Netherlands; 14 Government of Saskatchewan, Disease Control Laboratory Regina, Canada; 15 Department of Bioengineering, University of California, San Diego, La Jolla, California, United States of America; 16 Centro de Biotecnologia, Instituto Butantan, São Paulo, SP, Brazil; 17 Programa Interunidades em Biotecnologia, Instituto de Ciências Biomédicas, USP, São Paulo, SP, Brazil; 18 Department of Medicine, University of Cambridge, Cambridge, United Kingdom; 19 Institut Pasteur, Biology of Spirochetes Unit, National Reference Centre and WHO Collaborating Center for Leptospirosis, Paris, France; 20 Instituto de Medicina Tropical Alexander von Humboldt; Facultad de Medicina Alberto Hurtado, Universidd Peruana Cayetano Heredia, Lima, Peru; 21 Faculty of Tropical Medicine, Mahidol University, Bangkok, Thailand; 22 Department of Microbiology and Immunology, Indiana University School of Medicine, Indianapolis, Indiana, United States of America; 23 Instituto de Medicina “Alexander von Humboldt,” Universidad Peruana Cayetano Heredia, Lima, Peru; University of Tennessee, UNITED STATES

## Abstract

Leptospirosis, caused by spirochetes of the genus *Leptospira*, is a globally widespread, neglected and emerging zoonotic disease. While whole genome analysis of individual pathogenic, intermediately pathogenic and saprophytic *Leptospira* species has been reported, comprehensive cross-species genomic comparison of all known species of infectious and non-infectious *Leptospira*, with the goal of identifying genes related to pathogenesis and mammalian host adaptation, remains a key gap in the field. Infectious *Leptospira*, comprised of pathogenic and intermediately pathogenic *Leptospira*, evolutionarily diverged from non-infectious, saprophytic *Leptospira*, as demonstrated by the following computational biology analyses: 1) the definitive taxonomy and evolutionary relatedness among all known *Leptospira* species; 2) genomically-predicted metabolic reconstructions that indicate novel adaptation of infectious *Leptospira* to mammals, including sialic acid biosynthesis, pathogen-specific porphyrin metabolism and the first-time demonstration of cobalamin (B12) autotrophy as a bacterial virulence factor; 3) CRISPR/Cas systems demonstrated only to be present in pathogenic *Leptospira*, suggesting a potential mechanism for this clade’s refractoriness to gene targeting; 4) finding *Leptospira* pathogen-specific specialized protein secretion systems; 5) novel virulence-related genes/gene families such as the Virulence Modifying (VM) (PF07598 paralogs) proteins and pathogen-specific adhesins; 6) discovery of novel, pathogen-specific protein modification and secretion mechanisms including unique lipoprotein signal peptide motifs, *Sec*-independent twin arginine protein secretion motifs, and the absence of certain canonical signal recognition particle proteins from all *Leptospira*; and 7) and demonstration of infectious *Leptospira*-specific signal-responsive gene expression, motility and chemotaxis systems. By identifying large scale changes in infectious (pathogenic and intermediately pathogenic) vs. non-infectious *Leptospira*, this work provides new insights into the evolution of a genus of bacterial pathogens. This work will be a comprehensive roadmap for understanding leptospirosis pathogenesis. More generally, it provides new insights into mechanisms by which bacterial pathogens adapt to mammalian hosts.

## Author Summary

Leptospirosis is an emerging and re-emerging globally important zoonotic infectious disease caused by spirochetes of the genus *Leptospira*. This genus is complex, with members that cause lethal human disease, yet mechanisms that underlie pathogenesis remain obscure. *Leptospira* species are divided into those that are infectious for mammals, and those that are non-infectious environmental saprophytes. Based on biological characteristics and molecular phylogeny, infectious *Leptospira* are further divided into pathogenic and intermediately pathogenic members. The pan-genus genomic analysis of 20 *Leptospira* species reported here shows the evolutionary relationship of the different *Leptospira* clades, and various genetic factors related to virulence and pathogenesis. Infectious *Leptospira* show key adaptations to mammals, for example sialic acid biosynthesis, pathogen-specific porphyrin metabolism, and the observation that pathogenic *Leptospira* are vitamin B12 autotrophs, able to synthesize it from a simple amino acid precursor, L-glutamine. A large novel protein family of unknown function—the Virulence Modifying proteins—is found uniquely in pathogenic *Leptospira*. Similarly, the CRISPR/Cas system was only found in pathogenic *Leptospira*. A comparative genomic analysis of a complex bacterial genus allowed us to identify large-scale changes that provides new insights into general processes by which bacteria evolve to become pathogenic.

## Introduction

Leptospirosis is a globally widespread zoonotic disease with important health consequences for humans and domesticated animals [1, 2]. Infectious *Leptospira* have significant affinity for specific mammals but vary in how strictly they adapt to specific hosts [1]. Rodent reservoirs (e.g., reservoir hosts (rats, mice) do not exhibit disease but have long-term renal colonization and excrete organisms in the urine, which is key to leptospiral ecology and its life cycle. Infected livestock (e.g.,. cattle, pigs) and companion animals (e.g.,. dogs) may suffer fetal loss and acute kidney, liver and lung injury in response to infection. Infected humans variably exhibit clinical manifestations including asymptomatic infection [3] with or without long-term renal carriage [4], undifferentiated fever, renal failure, jaundice, hemorrhage (especially the severe pulmonary hemorrhage syndrome), meningitis, shock and death.

Past taxonomy divided the *Leptospira* genus into a single pathogenic and a single saprophytic species denoted as *L*. *interrogans* and *L*. *biflexa*, respectively, which, in turn, were divided into more than 250 serovars based on the cross-agglutinin absorption (CAAT) assay [1, 5]. In the 1990s, DNA hybridization (DDH) identified 17 ‘genomospecies’ [6], which also distinguished DDH from serovar. DDH complemented by molecular methods and experimental studies have since confirmed the existence of at least 22 species [7–12], and grouping of species as infectious (sometimes referred to as group I and group II pathogens, corresponding to “pathogenic” and “intermediately pathogenic”, respectively) and non-infectious (“saprophytic”) [13]. Technical challenges in performing DDH has led to the development of many different molecular approaches to species identification [14, 15]. The International Committee on Systematics of Prokaryotes, Subcommittee on the Taxonomy of *Leptospiraceae* recently agreed that genome sequence comparison should replace DDH for species definition [16]. Such methods include sequence-based phylogeny and calculation of *in silico* genomic similarities between isolates by using draft genomes [17–20].

Leptospiral typing is important for carrying out outbreak investigations and in identifying likely mammalian host reservoir sources of infection. Two commonly used molecular methods performed are pulsed-field gel electrophoresis and multilocus sequencing typing (MLST). MLST has the advantage that it reflects the underlying population genetic structure, is reproducible, is robustly supported by experimental data, and even can be used directly to identify infecting *Leptospira* in clinical samples [21–27]. Genome sequencing, which has become widely available, together with automated tools that assign MLST sequence types directly from sequence data, has demonstrated an important potential for typing, with the expectation that automated analysis tools will become sufficiently user-friendly for rapid and efficient whole genome analysis and comparison, including phylogenetic analysis based on the identification of single nucleotide polymorphisms (SNPs) in the core genome.

The goal of the *Leptospira* Genome Project, initiated in 2011, has been to obtain and compare whole genome information for all known *Leptospira* species. Among the goals of this analysis are the following: i) identifying *Leptospira* pathogenesis mechanisms that might explain heterogeneity in clinical manifestations of leptospirosis; ii) understanding the relationship of genomic content and context to pathogenesis; iii) determining the definitive evolutionary relationship of *Leptospira* towards understanding how infectious *Leptospira* diverged from saprophytes; and iv) identifying common antigens for improving diagnosis and vaccine development. Prior to this project, there were 9 known pathogenic *Leptospira* species, 5 intermediate *Leptospira* species, and 6 saprophytic *Leptospira* species [28], for which whole genome sequence analysis was available for two pathogenic species (two serovars of *L*. *interrogans*, Lai *[29]* and Copenhageni [30], two serovars of *L*. *borgpetersenii* [31]), one intermediate pathogen *L*. *licerasiae* [32], and one saprophyte species, *L*. *biflexa* [33]. Since the advent of the present large-scale project the whole genome sequence of another pathogen, *L*. *santarosai* serovar Sherman has been reported [34] but without comparative analysis.

The present study reports a systematic comparative genome analysis of the 20 *Leptospira* species known when this project began. These species comprise the pathogenic, intermediately pathogenic and saprophytic clades, defined by 16S rRNA gene sequence [2, 35, 36] and complemented by DNA-DNA hybridization [7, 11, 32]. This analysis focuses on the main genomic features and content distinguishing infectious from non-infectious *Leptospira*, and on how specific genes and gene families have ramified in the pathogenic and intermediately pathogenic *Leptospira*.

## Methods

### *Leptospira* isolates

A globally representative collection of the 20 *Leptospira* species known at the advent of this project was analyzed here, and provided by members of the leptospirosis research community (Table 1).

**Table 1:** Leptospiral species metadata and genome statistics.

Species	Serovar	Strain	Lifestyle	Source	Country of Origin	Contributing Lab	Bioproject	WGS Master or GenBank Accession	BEI/ATCC	Size (Mbp)	# Contigs	#genes	#proteins	Contig N50	#Scaffolds	Average Coverage	G+C%	Locus Tag	Finishing status^§^
*alexanderi*	Manhao 3	L 60^T^	pathogenic	*Homo sapiens*	China	Hartskeerl	PRJNA74069	AHMT00000000.2	NR-22256	4.22	65	4582	4541	156,869	14	58.1x	40.2%	LEP1GSC062	IHQD
*alstoni*	Pingchang	80–412	pathogenic	Frog	China	Galloway	PRJNA177154	AOHD00000000.2	NR-35361	4.44	67	4392	4380	152,980	33	57.6x	42.5%	LEP1GSC193	IHQD
*borgpetersenii*	Javanica	UI 09931	pathogenic	*Homo sapiens*	Thailand	Peacock	PRJNA74113	AHNP00000000.2	NR-20171	3.89	15	4053	4010	468,398	8	60.4x	40.1%	LEP1GSC103	IHQD
interrogans*	Copenhageni	Fiocruz L1-130	pathogenic	*Homo sapiens*	Brazil	-	PRJNA10687	AE016823/4	ATCC BAA-1198	4.63	2	3762	3667	-	2	-	35.0%	LIC	Complete
*kirschneri*	Cynopteri	3522 C^T^	pathogenic	Chiroptera (bat)	Indonesia	Hartskeerl	PRJNA74057	AHMN00000000.2	NR-22255	4.41	24	4029	3986	415,138	13	59.0x	35.9%	LEP1GSC049	IHQD
*kmetyi*	Malaysia	Bejo-Iso9^T^	pathogenic	environmental	Malaysia	Hartskeerl	PRJNA74061	AHMP00000000.2	NR-22254	4.42	4	4282	4238	3,808,637	4	57.2x	44.8%	LEP1GSC052	IHQD
*noguchii*	Panama	CZ 214^T^	pathogenic	*Didelphis marsupialis* (opossum)	Panama	Hartskeerl	PRJNA167231	AKWY00000000.2	NR-22283	4.71	35	4565	4520	280,299	12	56.2x	35.5%	LEP1GSC059	IHQD
*santarosai*	Shermani	1342K^T^	pathogenic	*Homo sapiens*	Panama	Hartskeerl	PRJNA178172	AOHB00000000.2	Not Deposited	3.99	68	4049	4002	108,025	40	62.8x	41.8%	LEP1GSC048	IHQD
*weilii*	undetermined	LNT 1234	pathogenic	*Homo sapiens*	Thailand	Peacock	PRJNA74087	AHNC00000000.2	NR-20183	4.26	83	4478	4436	172,307	24	59.7x	40.8%	LEP1GSC086	HQD
*broomii*	Hurstbridge	5399^T^	intermediate	*Homo sapiens*	Denmark	Hartskeerl	PRJNA74059	AHMO00000000.2	NR-22253	4.40	12	4249	4205	2,457,338	6	54.0x	43.0%	LEP1GSC050	IHQD
*fainei*	Hurstbridge	BUT 6^T^	intermediate	*Sus scrofa* (wild boar)	Australia	Hartskeerl	PRJNA167230	AKWZ00000000.2	NR-22252	4.29	13	4157	4113	579,278	5	59.1x	43.5%	LEP1GSC058	IHQD
*inadai*	Lyme	10^T^	intermediate	*Homo sapiens*	USA	Hartskeerl	PRJNA74055	AHMM00000000.2	NR-22251	4.46	27	4314	4264	810,442	14	55.9x	44.6%	LEP1GSC047	IHQD
*licerasiae*	Varillal	VAR 010^T^	intermediate	*Homo sapiens*	Peru	Vinetz	PRJNA74167	AHOO00000000.2	ATCC BAA-1110	4.21	14	3974	3931	553,148	4	58.3x	41.1%	LEP1GSC185	HQD
*wolffii*	Khorat	Khorat-H2^T^	intermediate	*Homo sapiens*	Thailand	Hartskeerl	PRJNA167232	AKWX00000000.2	NR-22250	4.40	23	4252	4206	916,783	10	58.5x	45.6%	LEP1GSC061	IHQD
*biflexa*^†^	Patoc	Patoc I (Paris)	saprophytic	stream water	Italy	-	PRJNA58993	CP000786/7	ATCC 23582	3.95	2	3774	3725	-	3	6x	38.9%	LEPBI	Complete
*meyeri*	Hardjo	Went 5	saprophytic	unknown	Canada	Galloway	PRJNA167225	AKXE00000000	NR-29052	4.19	7	4011	3969	2,478,877	3	61.1x	38.0%	LEP1GSC017	HQD
*terpstrae*	Hualin	LT 11–33^T^	saprophytic	unknown	China	Hartskeerl	PRJNA178717	AOGW00000000.2	ATCC 700639	4.09	23	3932	3889	372,285	10	67.1x	38.2%	LEP1GSC203	HQD
*vanthielii*	Holland	WaZ Holland	saprophytic	water	Netherlands	Galloway	PRJNA177160	AOGY00000000.2	ATCC 700522	4.23	89	4251	4205	164,554	28	58.1x	38.9%	LEP1GSC199	HQD
*wolbachii*	Codice	CDC	saprophytic	unknown	USA	Galloway	PRJNA177156	AOGZ00000000.2	NR-35357	4.08	25	3956	3911	2,540,234	9	70.5x	39.2%	LEP1GSC195	IHQD
*yanagawae*	Saopaulo	Sao Paulo^T^	saprophytic	water	Brazil	Hartskeerl	PRJNA178716	AOGX00000000.2	ATCC 700523	4.06	47	4011	3967	330,086	19	60.4x	38.2%	LEP1GSC202	IHQD

^**§**^ Improved High-Quality Draft (IHQD); High-Quality Draft (HQD)

*J. Bacteriol. 186 (7), 2164–2172 (2004)

^†^PLoS ONE 3 (2), E1607 (2008)

^T^type strain

### DNA preparation from isolates

A standard operating procedure was established for all contributing laboratories to follow in preparing DNA for whole genome sequencing. *Leptospira* were considered to be like Gram-negative bacteria for the purpose of DNA extraction because of the presence of lipopolysaccharide and a thin peptidoglycan cell wall. Either ~10^12^ bacterial cells or 30 mL of the densest possible culture of *Leptospira* (in EMJH medium) were centrifuged and the pellet resuspended in 180 μl Buffer ATL (all buffer abbreviations are according to the manufacturer and related specifically to components of the kit) (Qiagen Tissue Kit, Valencia, CA, USA), and then purified according to the protocol “Purification of Total DNA from Animal Tissues (Spin-Column Protocol),” including the use of proteinase K, thorough vortexing throughout the procedure, and incubated at 56°C until the cells were completely lysed, according to the manufacturer’s instructions. Lysis was usually complete in 1–3 hr. Before adding Buffer AL, 10 μl of RNAse cocktail was added (mixture of two highly purified ribonucleases, RNase A (500 U/ml) and RNase T1 (20,000 U/ml); Ambion, Life Technologies, Carlsbad, CA), and incubated for 30–60 min at 37°C. After vortexing for 15 s, Buffer AL was added to the sample, which was again mixed thoroughly by vortexing, followed by adding ethanol (96–100%), and mixed again by vortexing. It was considered essential that the sample, Buffer AL, and ethanol were mixed immediately and thoroughly by vortexing or pipetting to yield a homogeneous solution. After this point samples were handled with large bore, genomic DNA-compatible tips. The mixture from step 3 (including any precipitate) was pipetted into the DNeasy Mini spin column placed in a 2 ml collection tube (provided), centrifuged at 6000 x *g* for 1 min and the flow-through discarded. The DNeasy Mini spin column was placed in a new 2 ml collection tube, and washed with 500 μl Buffer AW1. The DNeasy Mini spin column was placed in a new 2 ml collection tube, washed with 500 μl Buffer AW2, and centrifuge for 3 min at 20,000 x g (14,000 rpm) to dry the DNeasy membrane; remaining ethanol was considered to interfere with sequencing reactions and processing. The DNeasy Mini spin column was eluted twice by adding 2 x 100 μl Buffer AE directly onto the DNeasy membrane, incubating at room temperature for 1 min, and then centrifuged for 1 min at 6000 x *g* (8000 rpm). DNA was shipped on dry ice to JCVI for sequencing. Quality control included certification of intact, high molecular weight DNA and required 15–30 μg for fragment libraries, complementarily documented by agarose gel image containing a DNA Mass Ladder, OD260/280 determination, and an estimated DNA concentration from a fluorometric assay (SYBR Green, Quant-IT PicoGreen dsDNA Assay Kits).

### Genome sequencing, draft assembly and annotation

The genomes of 17 *Leptospira* species (the whole genome sequences of the remaining 3 species studied in the present analysis, *L*. *interrogans* serovar Copenhageni strain L1-130 and *L*. *biflexa* serovar Patoc Strain Patoc I, and *L*. *licerasiae* serovar Varillal strain VAR010, already were published [33, 37, 38]) were sequenced at JCVI by a combination of Illumina HiSeq (2x100 bp) and 454 FLX Titanium. Briefly, paired-end libraries were constructed for each sequencing technology from random nebulized genomic DNA in the 300–800 bp (Illumina) and 2–3 kb (454) size ranges. Sequence reads were generated with a target average read depth of ~ 20–30 fold (454) and ~60-fold (Illumina) coverage. Sequences for all 18 strains were assembled using the Celera Assembler version 6.1 [38], and ordered using NUCmer [39] to align the contigs to the best-matching closed *Leptospira* reference genome. All 18 new genome sequences underwent manual gap closure to elevate the genome status to improved high-quality draft (Table 1). Contigs were annotated for protein- and RNA-encoding features using the JCVI automated annotation pipeline essentially as described [40–43] except HMMs were run using HMMER3 [44].

### Phylogenetic analysis

16S rRNA trees were generated by first creating a multiple sequence alignment to the bacterial 16S rRNA reference alignment using Ribosomal Database Project release 10 (RDP-X) [45]. The aligned FASTA sequences were downloaded and trimmed to remove gapped columns using Belvu (v2.31) [46]. Based on the alignment, a bootstrapped Maximum-likelihood tree was subsequently inferred using phylipFasta, an in-house wrapper script [47] for the Phylip program [48, 49].

SecY trees were created by first aligning *secY* nucleotide sequences using Clustal Omega [50] with 100 combined guide-tree/HMM iterations. The multiple sequence alignment was trimmed to remove gapped columns and a bootstrapped Maximum-likelihood tree was inferred as was done for the 16S rRNA trees.

The nucleotide sequences of 7 MLST housekeeping genes were extracted from the 20 genomes, and sequence types (STs) assigned using the MLST website (http://leptospira.mlst.net/) [51]. A multiple sequence alignment of the concatenated sequences of 7 MLST loci was performed using ClustalW implemented in MEGA version 5 [52]. A maximum likelihood tree was re-constructed using an algorithm implemented in PhyML version 3.0.1 [53]. The model of sequence evolution used was the generalized time-reversible (GTR) model with gamma-distributed rate variation. The CLC Main Work Bench version 7.0 was used to edit and display the tree (Qiagen, USA).

Universal protein marker trees were constructed using a set of 39 proteins that are universally conserved among bacteria and produce monophyletic phylogenies, suggesting that they undergo minimal horizontal transfer (S1 Table) [54–56]. Protein sequences were aligned using ClustalW (v1.83) [57] using default settings. The alignment was trimmed to remove gapped columns using trimAl (v1.2r59) using–nogaps option and–fasta output option [58]. Aligned and trimmed predicted amino acid sequences of each species were concatenated as described previously [40], in the following order: AspS, FusA, GyrB, InfB, LepA, LeuS, PyrG, RplA, RplB, RplC, RplE, RplF, RplK, RplM, RplN, RplO, RplP, RplR, RplV RpoA, RpoB, RpsB, RpsC, RpsD, RpsE, RpsG, RpsH, RpsI, RpsK, RpsL, RpsM, RpsO, RpsQ, SecY, SerS, TopA, TsaD, Tuf, and YchF. The resulting alignment of 11241 amino acids was used to generate a Maximum-likelihood tree from 100 bootstrapped replicates using raxmlFasta, an in-house wrapper script for the raxmlHPC (v7.0.4) [59].

A pan-genome tree was constructed using the mean of the BLASTP Score Ratio (BSR) as described previously [60]. The PanOCT output file *100_pairwise_BSR_distance_matrix_phylip*.*txt* was used as input for Neighbor [48, 49] to build an unrooted UPGMA Neighbor-Joining tree. This PanOCT output file is a Phylip-style distance matrix derived from the pairwise mean BSR of core proteins present in 100% of genomes where a single value is presented for each pair of genomes in the pan-genome.

### *In silico* DNA-DNA hybridization

Genome relatedness among *Leptospira* strains was determined pairwise from fully or partially sequenced genomes using the genome-to-genome distances (GGD) calculator (S2 Table) [61]. This analysis was complemented by *in silico* DNA-DNA hybridization as previously reported [11].

### Pan-genome analysis

Clusters of orthologous proteins were generated using version ver3_13 of PanOCT [62]. Since PanOCT does not place paralogs into its ortholog clusters, but does produce a paralogs.txt file that specifies which clusters are paralogs, an in-house PERL script, paralogs_matchtable.pl, was created to merge paralogous clusters. This approach was necessary because analysis of core and novel genes has historically been defined for clusters containing all paralogs [63–68]. The R script, compute_pangenome.R, from Park et al. [67] and paralog_matchtable.pl were used to construct the pan-genome, core and novel genes plot. We initially chose not to compute permutations in genome order for the reasons described in [69]. As a consequence of a lack of permutations, compute_pangenome.R was modified to load in a defined genome order of addition.

Orthologous protein content was compared and illustrated in a Venn diagram that was constructed using output from an in-house PERL script, create_meta_groupings.pl that uses output from PanOCT and a file that describes how genomes are to be grouped. Genomes were grouped by whether they are infectious (group I or group II), non-infectious (saprophytic), or an outgroup (*Leptonema illini*). Since there were multiple genomes per group (except for *Leptonema*), clusters were counted if there was a majority (50%), all-but-one, or all protein members from a particular group or groups. Clusters not matching these criteria were not counted.

### Metabolic reconstructions

Four draft metabolic network reconstructions were created for representative species chosen from pathogenic, intermediate and saprophytic clades including the following: *L*. *interrogans* (serovar Copenhageni strain L1-130), *L*. *licerasiae* (serovar Varillal strain VAR010), *L*. *biflexa* (serovar Patoc strain Patoc 1 (Ames)) and *L*. *kmetyi* (serovar Malaysia strain Bejo-Iso9). *L*. *kmetyi* was included in these comparisons because in addition to having recently been reported to infect humans in the Caribbean islands [70, 71], initial genome examination suggested that *L*. *kmetyi* could belong to a transitional group between the group I and group II pathogens and distinct from the other group I pathogens, here represented by *L*. *interrogans*. The reconstructions were built using the ModelSeed framework [72].

The COBRApy toolbox [73] was used to perform Flux Balance Analysis (FBA) [74] simulations and constraint-based analyses using the gurobi linear programming solver [75]. The constraint-based model consists of an S matrix composed of distinct metabolites and reactions including exchange and biomass reactions (S3 Table). Each of the reactions has an upper and lower bound on the flux it can carry. Reversible reactions have an upper bound of 1,000 mmol gDW^−1^h^−1^ and a lower bound of −1,000 mmol gDW^−1^ h^−1^, making them practically unconstrained, while irreversible reactions have a lower bound of zero. By default, the biomass reaction was set as the objective to be maximized. The exchange reactions that allow for extracellular metabolites to pass in and out of the system were defined such that a positive flux indicates flow out. The GapFind MILP algorithm [76] encoded in the COBRApy Toolbox was performed for the models unable to grow in minimal medium (biomass objective function equal to zero), to find exchange reactions allowing for *in vitro* growth, indicating strain-specific auxotrophies.

An *in silico* minimal medium was constructed that supported growth for all of the *Leptospira* models, consisting of trace elements (magnesium, manganese zinc, sulfate, calcium, copper, phosphate, cobalt, chlorine, potassium, ferrous and ferric iron and ammonia), water, oxygen, heme, CO_2_, the vitamins thiamine (B1), folate and menaquinone, glycerol and the fatty acids lauric acid, stearic acid, and decanoate and aminoethanol, meso-2,6, diaminopimelate, as well a variety of amino acids (glutamate, aspartate, tyrosine, phenylalanine, asparagine (S3 Table).

### Genomic location and genetic organization of vitamin B12 biosynthesis -related gene clusters in *Leptospira*

PanOCT data were used to identify the cob I/III and cob II gene clusters in infectious and non-infectious *Leptospira* (when present), which encode proteins predicted to participate in B12 transport or synthesis. To determine the genomic locations of the *btuB* and cob II and I/III clusters, a custom *Postgresql* database was created using the annotated genomes of 20 species. Orthologs were identified with blastp (-F “m S” -s T) and conserved genomic neighborhood using the Prokaryotic Sequence homology Analysis Tool (PSAT) [77].

## Results

### General genomic descriptions

The genomes of 17 *Leptospira* spp. isolates were newly determined for this study, representing 8 pathogenic, 4 intermediate and 5 saprophytic clades and used in pan-genomic comparative analyses along with the previously reported genomes of *L*. *interrogans* serovar Copenhageni strain Fiocruz L1-130 [30], *L*. *biflexa* serovar Patoc strain Patoc I (Paris) [33] and *L*. *licerasiae* serovar Varillal strain VAR010^T^ [32]. Thirteen of these isolates were sequenced to a genome finishing status [78, 79] of "Improved High-Quality Draft" (IHQD) and 5 to a status a “High-Quality Draft" (HQD) (Table 1). To achieve a genome finishing status of IHQD, manual finishing was conducted consisting of contig sequence extension, sequence gap closure, and PCR to link physical ends. On average, the genomes assembled into 36 contigs [range 4 (*L*. *kmetyi*) to 89 (*L*. *vanthielii*)], with an average genome size of 4.26 Mbp in length [range 3.89 Mbp (*L*. *borgpetersenii* to 4.71 Mbp (*L*. *noguchii*)] at an average of 59.7-fold sequence coverage. The average G+C% was 40.7% [range 35.5% (*L*. *noguchii*) to 45.6% (*L*. *wolfii*)]. These genomes were predicted to encode an average of 4,197 protein-coding sequences per genome [range 3,932 (*L*. *terpstrae*) to 4,582 (*L*. *alexanderi*)].

### Phylogenetic analysis of the *Leptospira* Genus to determine evolutionary relatedness

**Fig 1:**
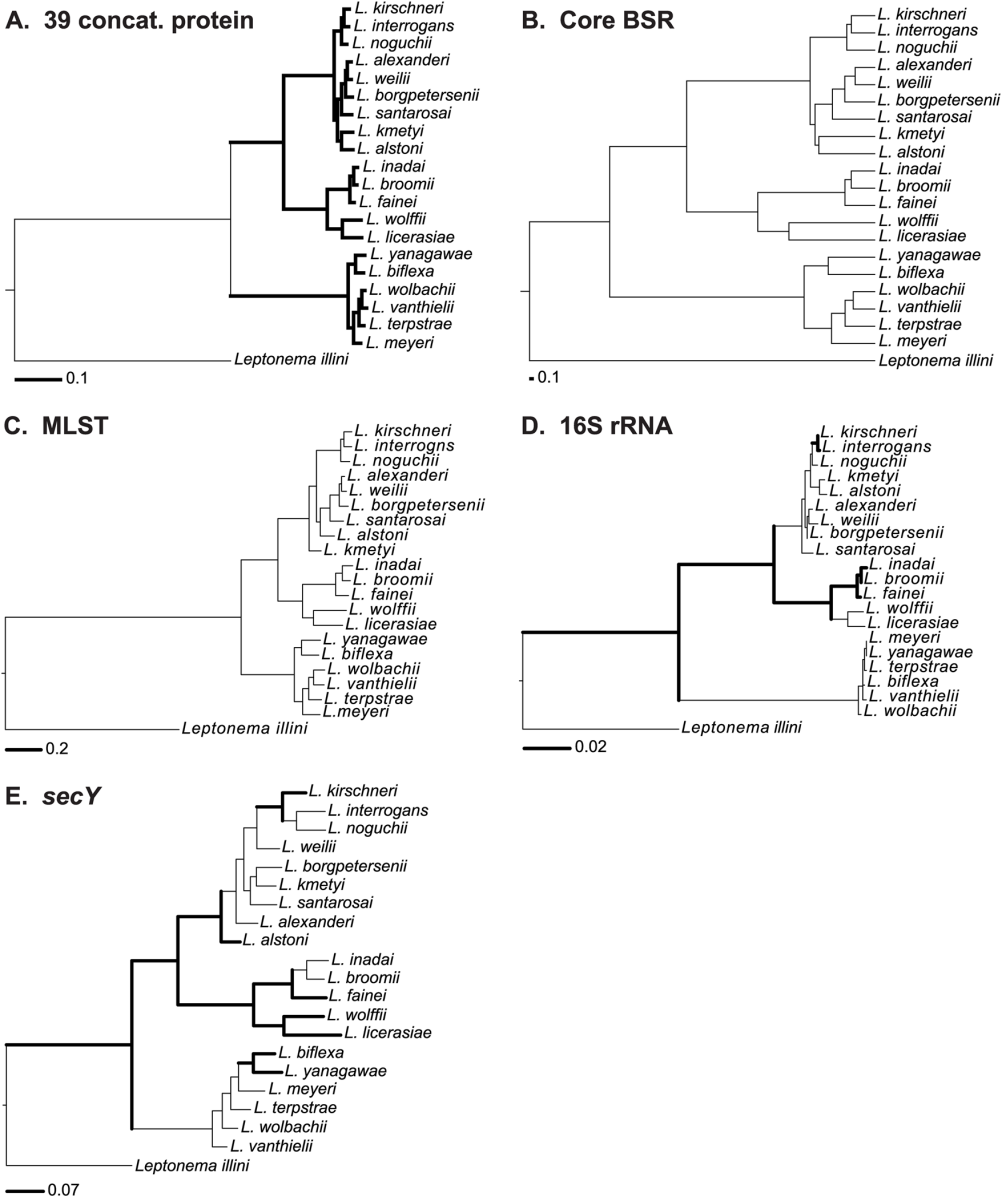
Phylogenetic analysis of *Leptospira* species. Consensus maximum-likelihood trees are depicted using multiple alignments of 16S rRNA (A), secY (B), MLST (C) and 39 concatenated protein data sets (D). The numbers along the branches denote percent occurrence of nodes among 100 bootstrap replicates. A pan-genome tree was generated based on the mean of the BLASTP score ratio of core 1135 proteins (E). The scale bar represents the number of nucleotide (A-C), amino acid (D & E) substitutions.

Twenty genome sequences (17 new, 3 previously published) of isolates representing 20 of the 22 known *Leptospira* spp. (Table 1) were used to determine phylogenetic distances between species; two recently reported species (*L*. *idonii* [80] and *L*. *mayottensis* [81]) are not included here. Phylogenetic relationships among all *Leptospira* species were analyzed in five independent ways (Fig 1): A, a core set of 39 concatenated genes coding for housekeeping proteins (universal markers); B, a pan-genus set of 1350 proteins; C, 3) multilocus sequence typing (MLST)[51]; D, 16S rRNA (highly conserved); and E, *secY* (highly variable)[70]. *Leptonema illini* was used as the outgroup for all analyses. Each approach yielded different nodes and branches of the species, except for 16S rRNA sequences, for which deduced phylogeny did not discriminate between *L*. *meyeri* and *L*. *yanagawae*. Additionally, these five approaches revealed three clades correctly clustering members of the nine pathogenic (group I) and five intermediately pathogenic (group II) and six non-pathogenic (saprophytic) species. Only the trees based on *secY*, the universal markers and the pan-genome clearly separated the closely related pathogenic species *L*. *interrogans*, *L*. *kirschneri* and *L*. *noguchii* from the other 6 pathogenic species. As expected, phylogenetic positions shifted to some extent between the single locus-based analyses and became more consistent using the multi-locus approaches. The following pairs of species showed close relationships: the pathogenic species (except in the *secY* tree) *L*. *interrogans* and *L*. *kirschneri*, *L alexanderi* and *L*. *weilii*; the group II species, *L*. *inadai* and *L*. *broomii*; and the non-pathogenic (except 16S and *secY* trees) *L*. *wolbachii* and *L*. *vanthielii*.

Furthermore, the genome relatedness between pairs of representative strains of each of the 20 species from fully or partially sequenced genomes confirmed the genetic relatedness among *Leptospira* species as established by DDH (Fig 1/ S2 Table).

### Pan-genome analysis

The pan-genome is defined as a core set of genes shared by all isolates plus a variable set of genes shared by a subset of isolates, and strain-specific or novel genes. Based on these 20 representative genomes and raw PanOCT output, the size of the core- and pan-genome was determined to contain 1,764 and 17,477 genes, respectively; however, with paralogs collapsed, the size of the core- and pan-genome was determined to be 1,592 and 13,822 genes, respectively (S1A Fig). The number of species-specific or novel genes ranged from 233 to 892 (S1A Fig) for each new genome added. After the addition of the third genome (*L*. *noguchii*), the size of the core gene set plateaued, while the pan-genome continued to rise (S1A Fig).

To determine whether the *Leptospira* pan-genome is open or closed (as defined below, the number of new genes identified (i.e., unique or strain-specific genes) for each genome added was determined and fit to a power law function (n = κN^-α^) as described previously [63]. Conceptually, a pan-genome is closed when sequencing the genomes of additional isolates fails to increase gene number (i.e., the entire gene repertoire has been discovered) [82]. The exponent (α) indicates whether the pan-genome is open (α ≤ 1) or closed (α > 1) [83]. Using this equation, the pan-genome of *Leptospira* was inferred to be open (α = 0.49 ± 0.02) (S1B Fig). From an exponential decay function, the number of new genes predicted for each genome (species) added was extrapolated and calculated to be 409 ± 12 on average (S1B Fig).

**Fig 2:**
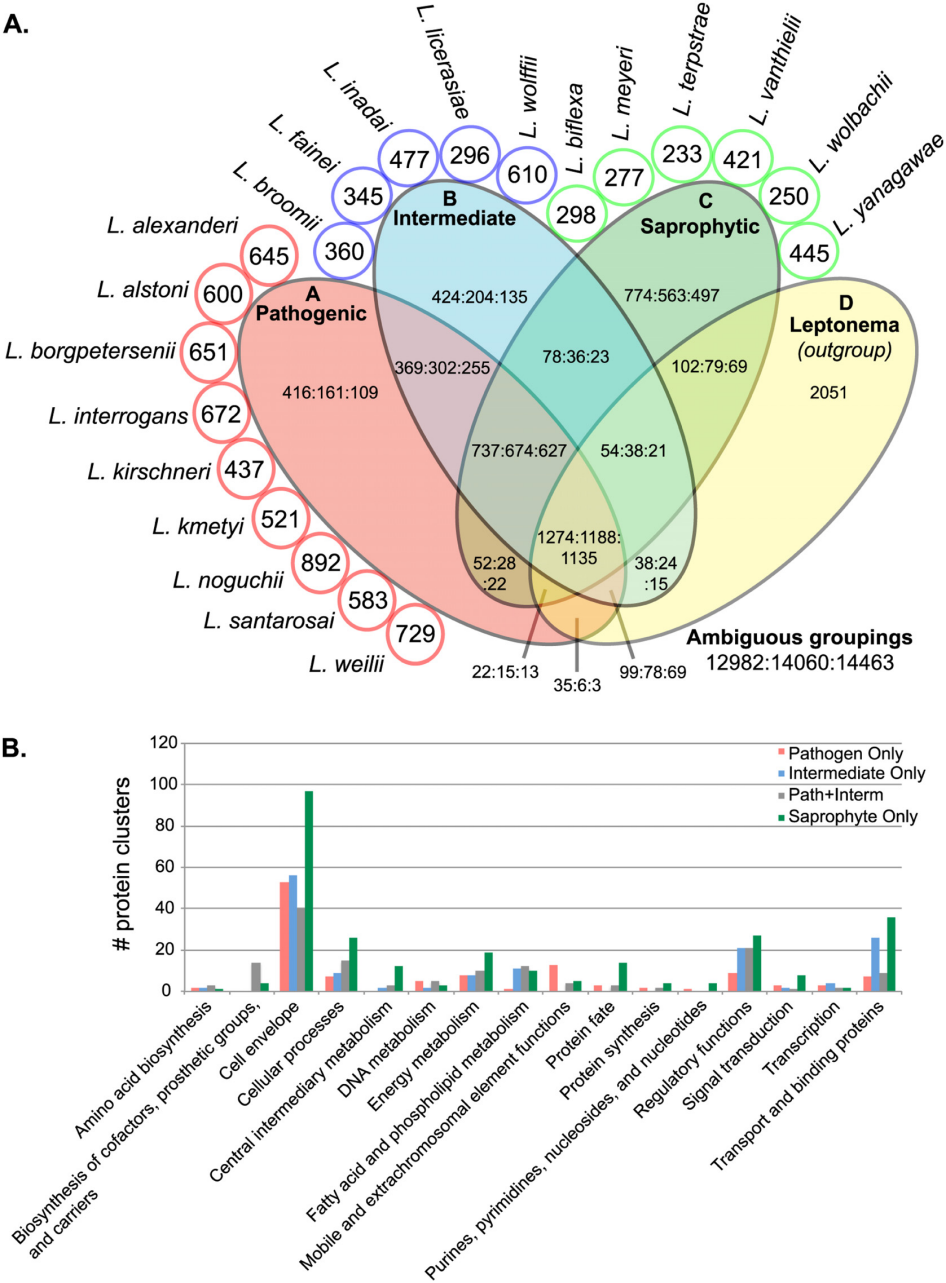
Pan-genomic comparisons of 20 *Leptospira* species. **Panel A:** Orthologous protein clusters were binned, counted and placed into a Venn diagram by whether clusters contained proteins from genomes in each of the three *Leptospira* groups: pathogenic (A), intermediate (B), saprophytic (C) and the *Leptonema* outgroup (D). Clusters were counted if there was a majority (50%), all-but-one, or all protein members from a particular group or groups (separated by colons). Singleton clusters, representing species-specific or strain-specific genes are noted in circles surrounding the Venn diagram. Clusters not matching any of these criteria or containing at least one protein from another group were considered as ambiguous groupings. The Venn diagram is not to scale. **Panel B:** Protein clusters unique to pathogenic, intermediate, and saprophytic groups or shared only between pathogenic and intermediate groups were counted by main functional role categories. See key for group colors.

The distribution of protein clusters representing gene families among the three groups (pathogenic, intermediate, saprophytic) is depicted in a Venn diagram (Fig 2A). Because there were multiple genomes per group (except for the *Leptonema* outgroup), clusters were counted if there was a majority (50%), all-but-one, or all protein cluster members from a particular group or groups. Focusing on the majority criteria, pathogens and intermediates had nearly equal numbers of group-specific genes (416 and 424, respectively), and the highest number of shared genes between two groups (369). Binary comparisons of pathogens and intermediates with saprophytes revealed just 52, and 78 genes shared, respectively. When comparing only *Leptospira*-specific genes, the core genome was comprised of 737 genes, with the majority of genes being shared with *Leptonema illini*. Closer examination of species-specific genes showed that pathogenic *Leptospira* have more species-specific genes on average (637±129) than do intermediates (418±126) or saprophytes (321±90). *L*. *noguchii* sv. Panama str. CZ 214T had the greatest number of species-specific genes among species compared in this study. To understand the function of genes shared among infectious *Leptospira*, the distribution of protein functions was examined for clusters shared among infectious and non-infectious *Leptospira* (Fig 2B). The only functional category dominated by pathogenic *Leptospira* was “mobile and extrachromosomal elements.” The functional categories that stood out most among genes shared between pathogens and intermediates was "biosynthesis of cofactors, prosthetic groups, and carriers" and "fatty acid and phospholipid metabolism." Saprophyte-specific genes dominated 10 of the 16 functional role categories, many of which were involved in central intermediary and energy metabolism, gene regulation, signal transduction, protein fate, cell envelope, and transport functions.

### Protein secretion systems

All *Leptospira* clades are predicted to have type II protein secretion systems but do not appear to contain a type III secretion system. Lipoproteins in *Leptospira* have particular pathogenetic significance because of their potential as vaccine targets and virulence factors involved in host-pathogen interactions [84–93].

### An unusual *sec* system in *Leptospira*

*Leptospira* contains the *Sec* system for signal-peptide-containing proteins and signal peptidase to remove the signal peptide at the time of secretion. Genes encoding the signal recognition particle (SRP) protein Ffh and receptor protein FtsY were not found in any *Leptospira* genome, nor was the SRP structural RNA. Generally, the lack of SRP and its receptor is unusual in bacteria, although the system is missing in the genus *Dehalococcoides* and also, apparently, in the uncultivated marine lineage SAR86 [94]. The narrow, elongated spiral shape limits the distance a ribosome can be from the *Leptospira* plasma membrane and may obviate the need for translation arrest by SRP. However, in looking for novel features near Sec system genes in *Leptospira* showed a novel gene inserted between the normally consecutive genes for Sec system proteins SecY and YajC, encoding a non-globular protein with an N-terminal signal peptide and a transmembrane segment towards the C-terminus, with the majority of residues in between consisting of low complexity, poorly conserved sequence especially rich in Lys, Glu, and Asn. No homologs to this low-complexity protein occur outside the *Leptospira* genus. We postulate that this novel gene could be involved in protein secretion.

### Unusual sec-independent (twin-arginine) translocation system

Twin-arginine translocation (TAT) in prokaryotes allows completion of complete protein folding prior to Sec-independent secretion through the plasma membrane. Except in the halophiles, where high salt outside the cell explains the need for folding prior to export, TAT substrates tend to be redox cofactor-binding proteins [95]. These proteins fold and bind their cofactors before crossing the membrane. The *tatA* and *tatC* gene-encoded components of the translocase are evident in *Leptospira* genomes, but the twin-arginine signal itself proved elusive. The TIGRFAMs collection [96] hidden Markov model (HMM) TIGR01409 finds no sequence scoring near the trusted cutoff in any species of *Leptospira*, nor in *Leptonema illini*. However, alignments of full-length homologs in *Leptospira* to recognizable TAT translocation substrates from other lineages could be extended into the N-terminal signal region. Such alignments often showed a Lys-Arg dipeptide in the *Leptospira* sequence aligned to the Arg-Arg motif of recognizable TAT signal sequences. This observation triggered a review of all candidate families of TAT translocation substrates in *Leptospira*, and produced iterative refinement of the lineage-specific TAT signal, and a catalog of TAT substrates.

**Fig 3:**
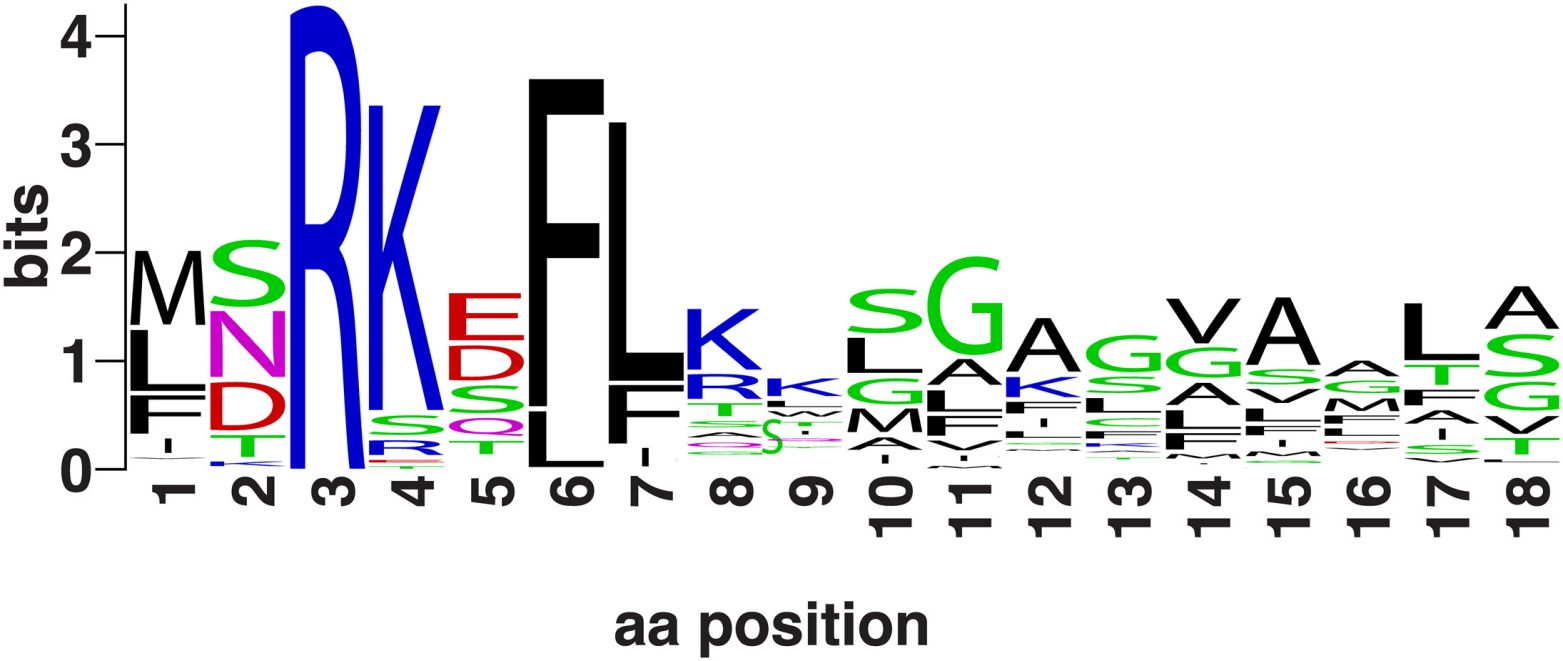
TAT signal sequence in *Leptospira sp*. The X-axis shows position in an ungapped alignment. The Y-axis shows information content, measured in bits.

Eleven protein families were confirmed as TAT substrates by multiple criteria, including strong conservation of the putative TAT signal within the protein family, alignment to non-spirochete homologs that extended N-terminally into the TAT signal region, and strong sequence similarity of the putative TAT signal motif, usually RKxFL, across the different *Leptospira* putative TAT translocation substrate families. A continuous 18-residue stretch from each protein in each of these was used to construct a seed alignment of *Leptospira* TAT signal sequences, including the modified Twin-Arg motif and the adjacent hydrophobic region. Comparative analysis predicted a conserved TAT signal sequence in all *Leptospira* [97] (Fig 3). Eleven protein families comprising the defining alignment and for two additional families are strong candidates for TAT-dependent, Sec-independent translocation (S4 Table). Only one of the 13 families, the PhoX alkaline phosphatase family, was observed to be largely restricted to pathogenic species of *Leptospira* [98].

Inspecting the family of LIC_10874 (a 4Fe-4S dicluster domain protein family of LIC_10874) within and outside the genus *Leptospira* demonstrated conservation of the putative TAT signal in both, and the substitution in *Leptospira* of the second Arg by Lys, as in other families. This family is notable, however, because in multiple species from phylogenetically distant clades, translation start sites can be assigned with high confidence, and the TAT signal begins rather far (some 50 residues) from that start. Member sequences in this family all share a well-conserved prefix domain, ~50 amino acids in length, between the start of translation, and star of the recognizable TAT signal.

An unexpected feature in the *Leptospira* TAT system cassette is a probable serine phosphatase encoded next to *tatC* (family TIGR04400), which either overlaps it or is present within five base pairs in all 20 leptospiral species examined. It is not known whether this putative phosphatase is involved in *Sec*-independent translocation *per se*, rather than in its regulation, or in some unrelated process.

### Lipoprotein secretion

Cleavage of pro-lipoproteins by the type II signal peptidase occurs within a short lipobox sequence, which includes the invariant cysteine that is targeted for covalent modification with lipids. In *E*. *coli* lipoproteins, the -1 position immediately preceding the peptidase cleavage site is highly conserved, being occupied by the small nonpolar amino acids Ala or Gly in the vast majority of cases [99]. A previous analysis noted somewhat larger residues, such as Asn, Ser, and Cys, at the -1 position of experimentally-verified spirochete lipoproteins [100]. Examination of *L*. *interrogans* lipoprotein orthologs in saprophytic and intermediate species revealed a number of unexpected amino acids at the -1 position (S5 Table). For example, the bulky amino acid Tyr was found in the -1 position in LipL21 in all intermediate *Leptospira* spp., whereas those from pathogenic and saprophytic species possess typical -1 residues Ala and Ser, respectively. Conversely, some lipoproteins of saprophytic or intermediate species with expected amino acids at the -1 position were found to have orthologs in *L*. *interrogans* with variant amino acids at the -1 position (S5 Table). For example, the outer membrane lipoprotein Loa22 of saprophytic species has the allowed residue Asn at -1 while its orthologs in pathogenic and intermediate species has Leu or Phe at the -1 position. Similarly, LIC11088 orthologs in most intermediate and saprophytic species possess permitted residues at -1, whereas the pathogens have Gln or charged residues. Thus, the availability of genome sequences from across the genus *Leptospira* has confirmed the much higher flexibility in the leptospiral lipobox and is anticipated to lead to redefinition of the pan-leptospiral lipobox to accommodate increased amino acid flexibility at the -1 position.

The substrate specificities of the first two enzymes in lipoprotein biogenesis, prolipoprotein diacylglyceryl transferase (Lgt) and signal peptidase II (Lsp), are likely to be influenced by amino acids at the -1 position relative to the lipoprotein cleavage site [101]. For this reason, these enzymes would be expected to possess novel structural properties that allow recognition of an expanded set of residues at the -1 position of the lipobox. Consistent with this notion, Lgt orthologs of all 20 *Leptospira* strains lack the signature sequence that defines most Lgt proteins (Prosite accession PS01311) [102]. Interestingly, *Leptonema illini*, the bacterium most closely related to *Leptospira* among sequenced organisms, harbors two Lgt paralogs: one quite close in sequence to leptospiral Lgt orthologs and another with signature sequence similar to Lgt of all other organisms. This arrangement suggests duplication of *lgt* in an ancestor common to *Leptospira* and *Leptonema*, with subsequent loss of the latter and functional divergence of the former to accommodate bulkier -1 lipobox residues. Similarly, Lsp of *Leptospira* species possesses an extra 22 or 24 residues at a position corresponding to a location within the second periplasmic loop of *E*. *coli* Lsp [103], which is missing from the Lsp sequence of other bacteria with well-characterized lipoproteins, including the spirochetes *B*. *burgdorferi* and *T*. *pallidum*. The sequence features of leptospiral Lgt and Lsp suggest the presence of novel structural features at the active sites of these enzymes consistent with variability at the -1 position of the leptospiral lipobox.

### Metabolic reconstructions

**Fig 4:**
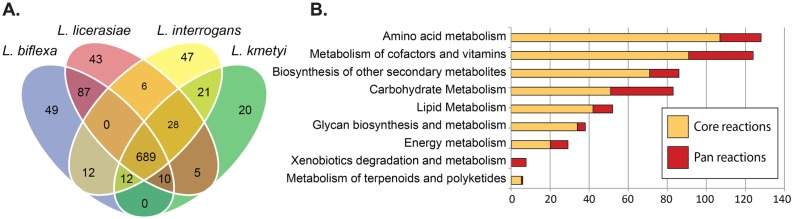
Core and pan-metabolic capabilities of the *Leptospira* genus. The core and pan metabolic content was determined for genome-scale metabolic models of 4 different *Leptospira* species. **A)** Core content, illustrated by the intersection of the Venn diagram, shared with all species. The pan content consists of all content in any model and includes the core content. The Venn diagram is not to scale. **B)** Classification of reactions in the core and pan reactomes by metabolic subsystem.

*In silico*, genomically-based metabolic network reconstructions were created for four representative *Leptospira* species occupying different clades: *L*. *interrogans* and *L*. *kmetyi* (pathogen; group I), *L*. *licerasiae* (intermediate pathogen; group II) and *L*. *biflexa* (non-pathogenic). *L*. *kmetyi* was chosen for analysis because preliminary genomic inspection suggested unusual features of this species with regard to pathogenesis-related genes (*vide infra*). The base *in silico* media and default computational bounds (S3 Table) represent every compound allowed to enter the system for cellular uptake to allow all models to produce biomass. Removal of some of these compounds leads to species-specific growth (a unique model-predicted auxotrophy). The use of a steady-state assumption does not allow the flux balance analysis to take into account specific concentrations of a given metabolite but, rather, the predicted rate of uptake, secretion or transformation. The default uptake bounds for each metabolite are provided (S3 Table). Negative bounds represent entry of the metabolite into the extra-cellular compartment where they can then be consumed by the model. The bounds are only constraints on the maximum rate of consumption of a given compound. The actual rate of consumption is predicted by the model. Units are in mmol gDW-1 hr-1. Analogously to core and pan genomes, the reaction content of each model can be used to construct core and pan metabolic networks. The core network consists of those reactions occurring in all representatives of *Leptospira*, while the pan network consists of all reactions that can potentially occur in any individual *Leptospira* species (Fig 4A).

Major differences in the metabolic networks between the four species arose in amino acid metabolism, biosynthesis of cofactors and vitamins and carbohydrate metabolism (Fig 4B). These large groups were further divided into specific pathways, specifically those of porphyrin metabolism, folate metabolism, starch and sucrose metabolism, as well as phenylalanine and tyrosine metabolism. A large difference was observed in porphyrin metabolism, specifically for the biosynthesis of cobalamin (vitamin B12) in *L*. *interrogans* (see below).

The conversion of static metabolic network reconstructions into computable mathematical models allows computation of phenotypes based on the content of each reconstruction [104]. Thus, the four strain-specific reconstructed networks were converted into genome-scale metabolic models that allow for the computational/simulation prediction of phenotype. This set of genomic scale models (GEMs) allows for a meaningful interpretation of the content of each reconstruction and allows for the prediction of each strain’s different metabolic capabilities [105]. Because reactions belonging to the amino acid metabolism subsystem made up the majority of reactions in the pan-reactome, it was hypothesized that these capabilities may reflect functional differences between different *Leptospira* species. To test this hypothesis, different minimal media formulations were created *in silico* and used to test each model’s growth capabilities. The models predicted all of the *Leptospira* tested to be auxotrophic for the amino acids aspartate, histidine and asparagine as well as vitamins B_1_ (thiamin) and vitamin K2 (menaquinone).

Beyond the auxotrophies predicted to be shared by all 4 *Leptospira* models, potential species-specific auxotrophies for other vitamins and amino acids were also identified. All of the strain-specific models were predicted to be auxotrophic for phenylalanine except for *L*. *interrogans*, which was predicted to have the enzyme prephenate dehydrogenase encoded for by *novF* that converts chorismate to prephanate, a precursor to tyrosine and phenylalanine. The model for *L*. *interrogans* lacks prephanate oxidoreductase, which would predict inability to convert prephanate to tyrosine. Only *L*. *kmetyi* was found to have the enzymatic machinery capable of synthesizing tyrosine from phenylalanine. Among these representative species of the three clades, only *L*. *interrogans* was predicted to be an L-glutamate auxotroph due to the lack of L-glutamate oxidoreductase.

Additional major differences were observed between the pathogenic and the non-pathogen *Leptospira*. A major difference in the lysine biosynthesis pathway was observed for the models of the pathogens, *L*. *kmetyi* and *L*. *interrogans*. Only these models possessed the *dapABCDE* genes required to convert L-aspartate 4-semialdehyde to LL-2,6, diaminopimelate required for peptidoglycan and lysine biosynthesis. Therefore both *L*. *licerasiae* and *L*. *biflexa* were predicted to be LL-2,6, diaminopimelate auxotrophs. Furthermore, only the pathogens *L*. *interrogans* and *L*. *kmetyi* possessed a full folate (vitamin B9) biosynthesis pathway using as precursor guanosine 5’-triphosphate. *L*. *biflexa* and *L*. *licerasiae* could produce vitamin B_2_ (riboflavin), but lack the reactions to convert it to folate including dihydroneopterin aldolase encoded for by *folB;* therefore, the models for *L*. *biflexa* and *L*. *licerasiae* were folate auxotrophs while the models for *L*. *kmetyi* and *L*. *interrogans* were not.

### Vitamin B12 biosynthesis

The vitamin B12 biosynthesis genes in infectious *Leptospira* are grouped into two clusters: cob I/III and cob II (Table 2). Though the exact number of reactions for each pathway in *Leptospira* remains unknown, in *Salmonella enterica* Typhimurium cob I comprises genes for the biosynthesis of adenosylcobinamide, cob II genes for the synthesis of the lower axial ligand 5,6-dimethylbenzimidazole (DMB) and a third cluster cob III, the nucleotide loop that joins DMB to the corrin ring to complete B12 biosynthesis. In infectious *Leptospira*, cob II is a five-gene cluster that includes three genes *cobTSC* that participate in the synthesis of DMB (and two genes that may or may not participate in B12 biosynthesis), suggesting that the first cluster encodes enzymes for the synthesis of adenosylcobinamide guanine diphosphate. The first 12 genes encode enzymes that participate in the synthesis of the corrin ring (cob I) whereas the last five, enzymes for the addition of the nucleotide loop (cob III). Intriguingly, cob III of the infectious species includes a gene *cbiZ* encoding an enzyme that participates in an alternative cobinamide salvage pathway first described in the archeon, strain Göl [106].

**Table 2:** Proteins involved in biosynthesis of vitamin B12 in *Leptospira*.

`				*L*. *interrogans* sv. Copenhageni str. Fiocruz L1-130	*L*. *kirschneri* sv. Cynopteri str. 3522 CT	*L*. *noguchii* sv. Panama str. CZ 214T	*L*. *alstoni* sv. Pingchang str. 80–412	*L*. *weilii* sv. undetermined str. LNT 1234	*L*. *alexanderi* sv. Manhao str. 3 L 60T	*L*. *borgpetersenii* sv. Javanica str. UI 09931	*L*. *santarosai* sv. Shermani str. 1342KT	*L*. *kmetyi* sv. Malaysia str. Bejo-Iso9T	*L*. *fainei* sv. Hurstbridge str. BUT 6T	*L*. *broomii* sv. Hurstbridge str. 5399T	*L*. *wolffii* sv. Khorat str. Khorat-H2T	*L*. *licerasiae* sv. Varillal str. VAR 010	*L*. *inadai* sv. Lyme str. 10T	*L*. *wolbachii* sv. Codice str. CDC	*L*. *yanagawae* sv. Saopaulo str. Sao Paulo	*L*. *biflexa* sv. Patoc str. Patoc1	*L*. *vanthielii* sv. Holland str. Waz Holland	*L*. *terpstrae* sv. Hualin str. LT 11-33T	*L*. *meyeri* sv. Hardjo str. Went 5
	Protein Name	Genbank Accession	Function	P	P	P	P	P	P	P	P	P	I	I	I	I	I	S	S	S	S	S	S
**cobI/III**	LB_149	NP_714693.1	phosphoglycerate mutase	+	+	+	+	-	+	-	+	+	+	+	-	+	+	-	-	-	-	-	-
	LB_150	NP_714694.1	cobalamin biosynthesis protein CobD/CbiB	+	+	+	+	-	+	+	+	+	+	+	-	+	+	-	-	-	-	-	-
	LB_151	NP_714695.2	histidinol-phosphate aminotransferase and cobyric acid synthase	+	+	+	+	-	+	+	+	+	+	+	-	+	+	-	-	-	-	-	-
	LB_152	NP_714696.2	cobinamide kinase	+	+	+	+	-	+	+	+	+	+	+	-	+	+	-	-	-	-	-	-
	LEP1GSC185_3352	EIE00386.1	histidine phosphatase superfamily (branch 1)	-	-	-	-	-	-	-	-	-	+	+	-	+	+	-	-	-	-	-	-
	LB_153	NP_714697.2	adenosylcobinamide amidohydrolase	+	+	+	+	-	+	+	+	+	+	+	-	+	+	-	-	-	-	-	-
	LB_154	NP_714698.1	cobyrinic acid a,c-diamide synthase	+	+	+	+	-	-	+	+	+	+	+	-	+	+	-	-	-	-	-	-
	LB_155	NP_714699.1	cob(I)yrinic acid a,c-diamide adenosyltransferase	+	+	+	+	-	+	+	+	+	+	+	-	+	+	-	-	-	-	-	-
	LB_156	NP_714700.1	precorrin-4 methylase	+	+	+	+	-	+	+	+	+	+	+	-	+	+	-	-	-	-	-	-
	LB_157	NP_714701.1	precorrin-3B C-17 methyltransferase	+	+	+	+	-	+	+	+	+	+	+	-	+	+	-	-	-	-	-	-
	LB_158	NP_714702.1	precorrin methylase	+	+	+	+	-	+	+	+	+	+	+	-	+	+	-	-	-	-	-	-
	LB_159	NP_714703.2	precorrin-2 C-20 methyltransferase	+	+	+	+	-	+	+	+	+	+	+	-	+	+	-	-	-	-	-	-
	LB_160	NP_714704.1	precorrin-6Y C5,15 methylase	+	+	+	+	-	+	+	+	+	+	+	-	+	+	-	-	-	-	-	-
	LB_161	NP_714705.2	cobalamin biosynthesis precorrin isomerase Cbic	+	+	+	+	-	+	+	+	+	+	+	-	+	+	-	-	-	-	-	-
	LB_162	NP_714706.1	cobalt-precorrin-6A synthase	+	+	+	+	-	+	+	+	+	+	+	-	+	+	-	-	-	-	-	-
	LB_163	NP_7147070.1	flavodoxin reductase	+	+	+	-	-	+	+	-	+	+	+	-	+	+	-	-	-	-	-	-
	LB_164	NP_714708.2	hypothetical protein	+	+	+	+	-	+	+	+	+	+	+	-	+	+	-	-	-	-	-	-
	LB_165	NP_714709.1	ferredoxin-related protein	+	+	+	+	-	+	+	+	+	+	+	-	+	+	-	-	-	-	-	-
	LEP1GSC185_3338	EIE00296.1	putative cobalt transporter subunit CbtA	-	-	-	-	-	-	-	-	-	+	+	-	+	+	-	-	-	-	-	-
	LEP1GSC185_3337	EIE00438.1	putative cobalt transporter subunit CbtB	-	-	-	-	-	-	-	-	-	+	+	-	+	+	-	-	-	-	-	-
**cobII**	LA_4202	NP_714382.1	putative lipoprotein	+	+	+	+	+	+	+	+	+	+	+	+	+	+	+	+	+	+	+	+
	LA_4203	NP_714383.1	putative lipoprotein	+	+	+	+	+	+	+	+	+	+	+	+	+	+	+	+	+	+	+	+
	LA_4204*	NP_714384.1	nicotinate-nucleotide—dimethylbenzimidazole phosphoribosyltransferase	+	+	+	+	+	+	+	+	+	-	-	+	+	-	+	+	+	+	+	+
	LA_4205	NP_714385.1	cobalamin synthase	+	+	+	+	+	+	+	+	+	+	+	+	+	+	+	+	+	+	+	+
	LA_4206**	NP_714386.1	phosphoglycerate mutase	+	+	+	+	+	+	+	+	+	+	-	+	+	-	+	+	+	+	+	+

P = Pathogenic species; I = Intermediate species; S = Saprophytic species

Cob I/III gene clusters in the sequenced pathogenic *Leptospira* vary in length, from 16 in *L*. *santarosai* Shermani 1342K^T^ to 19 in *L*. *alexanderi* Manhoa 3L60^T^ and *L*. *borgpetersenii* Javanica UI 0993. Presumably owing to repeated *in vitro* sub-culture in medium containing B12, several genes have been inactivated or deleted in the strains tested. For example, the Javanica UI 0993 cobI/III cluster contains a gene fragment resulting from a premature stop codon in a gene encoding a histidine phosphatase superfamily branch 1 (*hps*_1) protein present in all other pathogenic *Leptospira* including other *L*. *borgpetersenii* strains (Hardjo L550 and JB197); and, cob I/III in *L*. *alexanderi* 3L 60T, contains a disrupted cobyrinic acid a,c-diamide synthase, inactivated by a frame shift mutation. A gene encoding a flavodoxin reductase present in cobI/III of other infectious has been deleted in *L*. *santarosai* 1342KT and *L*. *alstoni* 80–412. The cob I/III clusters of *L*. *kmetyi* (group I) and all group II pathogens contain three genes, two encoding a putative cobalt transporter (*cbtBA*) and a gene encoding an additional *hps_1* protein (Table 2).

Cob II also varies in length among all infectious (pathogenic and intermediately pathogenic) *Leptospira*, from three genes in *L*. *broomii* Hurstbridge 5399 and *L*. *inadai* Lyme 10^T^ to seven in *L*. *noguchii* Panama CZ214^T^. The genes comprising the cob II cluster in non-pathogenic *Leptospira* are found in two discrete clusters in non-pathogenic species (e.g., LEPBI_I2857 and LEPBI_I2858, LEPBI_I2938 –LEPBI_I2940) in *L*. *biflexa* (Table 2), suggesting that homologs in pathogenic *Leptospira* were acquired *en bloc* after the divergence of pathogenic and non-pathogenic *Leptospira*.

### Leptospiral glycobiology: structure and diversity of *rfb*/O-antigen loci, lipid A, and sialic acid biosynthesis-encoding regions

#### General features of *Leptospira rfb* loci

Lipopolysaccharide (LPS) has long been a major focus of leptospiral microbiology not because of its (low potency) endotoxigenic activities (see below in the lipid A section) and because, notably, leptospiral LPS is the basis for serovar identification and vaccine development [36, 107–109]. Because of the importance of LPS in leptospiral biology, we carried out a comprehensive analysis of the genomic locations, structures and neighborhood of leptospiral *rfb* loci, also known as the O-antigen loci, in 20 species of *Leptospira*.

**Fig 5:**
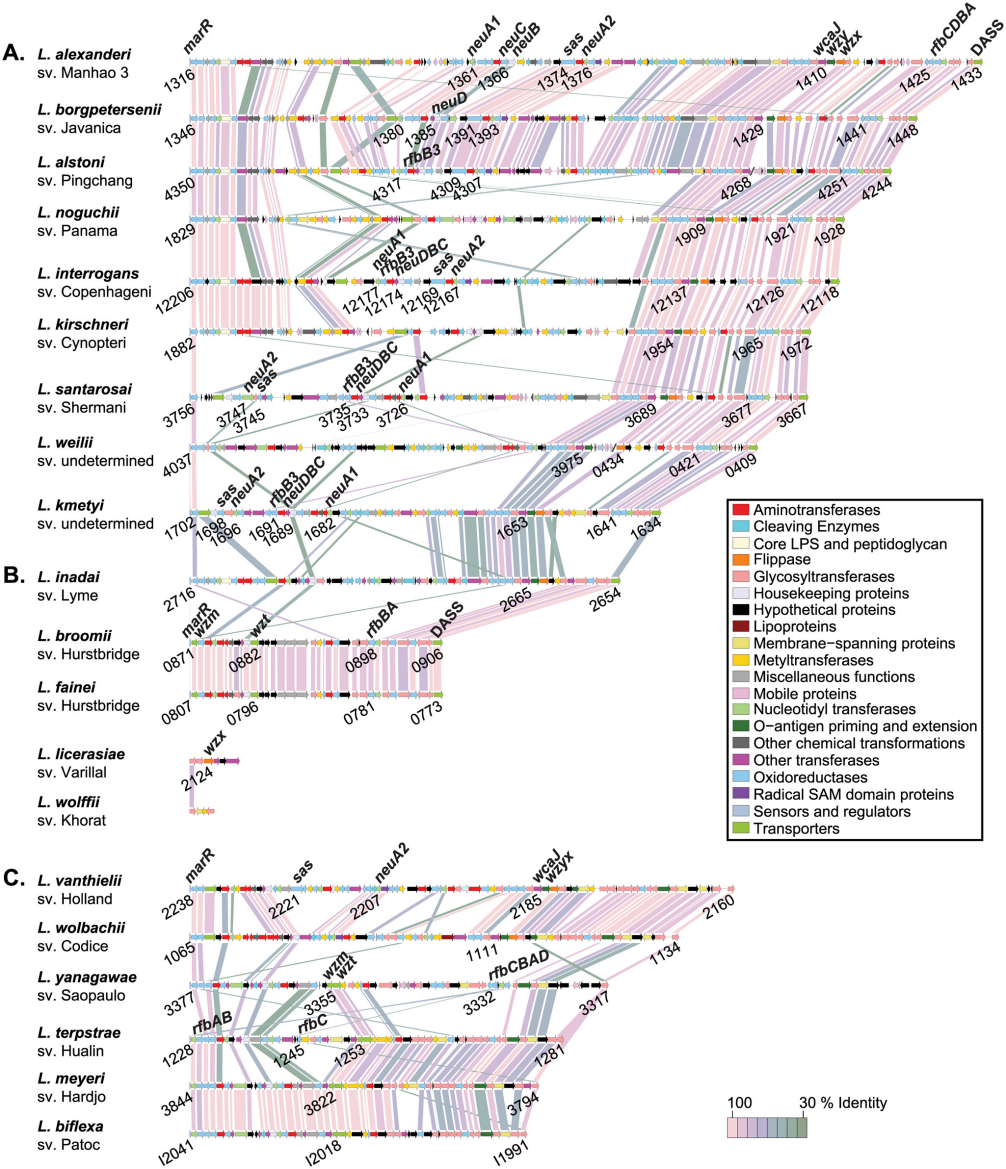
Structure of *Leptospira rfb locus* gene clusters. The *rfb* region and beginning and ending CDSs (blue) 9 of pathogenic (A), 5 intermediate (B), and 6 saprophytic (C) representative *Leptospira* species were compared. *rfb* region CDSs are labeled by locus identifier and colored by functional role categories as noted in the boxed key. Gene symbols, when present, are noted above their respective genes. BLASTP matches between CDSs are colored by protein percent identity (see key).

Using previously described leptospiral O-antigen gene clusters as a guide [110–112], we identified and schematized all clades of *Leptospira rfb* loci (Fig 5; S2 Fig depicts the *rfb* locus in genomic context). Of the genomes representing 20 *Leptospira* species, 17 known serovars were compared. The O-antigen biosynthesis gene clusters were located in three different genomic locations and ranged in size from 3,768 bp (*L*. *wolffii* sv. Khorat) to 121,402 bp (*L*. *alexanderi* sv. Manhao3). This region in *L*. *wolffii* sv. Khorat is now the smallest predicted leptospiral *rfb* biosynthesis cluster, consisting of just 4 genes, replacing the locus of *L*. *licerasiae* sv. Varillal [32]. All pathogenic leptospiral species, and the intermediates *L*. *inadai*, *L*. *broomii* and *L*. *fainei* have their *rfb* loci located in the same genomic position, sandwiched between a copper-binding protein on the left and the ribosomal protein S6 on the right (S2 Fig). The same protein-encoding genes (viz., *MarR* and *DASS*) define the start and end of the O-antigen cluster, respectively. Of the *rfb* loci of pathogenic *Leptospira*, serovars Manhao 3, Javanica, and Pingchang were most similar in size and gene content (Fig 5). Notably, *L*. *broomii* [113] (and *L*. *fainei* [114–116], both of which are serovar Hurstbridge, had nearly identical *rfb* gene clusters, predicting that *L*. *broomii* would also be serovar Hurstbridge, and confirmed by serology [113]. The presence of a specific serovar in different species has been previously observed in isolates of both *L*. *interrogans* and *L*. *borgpetersenii* serovar Hardjo, which have highly similar gene content in their O-antigen biosynthetic loci [117]. The *rfb* gene cluster of saprophytic *Leptospira* is downstream of the gene encoding ribosomal protein S6, lacks DASS, and is smaller (median 60,710 bp vs. ~99,520 bp) than pathogenic *Leptospira rfb* gene clusters (S2 Fig). O-antigen gene loci in serovars Varillal and Khorat are located in a third location, between *murC* and *purK*, consistent with a novel branching in the phylogenetic tree.

Consistently, the downstream flanking genes in the *rfb* loci are far more conserved than the upstream genes (Fig 5; S2 Fig). This finding is especially true for the pathogenic serovars and between three of the five intermediate serovars represented. For pathogenic serovars and serovar Lyme, a conserved block of genes is involved in O polysaccharide processing via the Wzy-dependent pathway. This export system was also identified in two saprophytic serovars (e.g., Holland and Codice). Overall, 12 of the representative 20 species genomes encoded the Wzy-dependent system and one genome (*L*. *licerasiae* sv. Varillal only encoded a putative flippase (Wzx) with no identifiable Wzy ortholog. There were no orthologs of Wzz, the O-antigen chain length determinant, in the 20 genomes studied. Also conserved in the 3-prime region of only those serovars with the Wzy-dependent pathway is a gene encoding a protein with homology to *E*. *coli WcaJ*/*S*. *enterica WbaP*, which are members of the PHPT family of polyisoprenyl-phosphate hexose-1-phosphate transferases that function to transfer glycosyl-1-phosphate to a lipid undecaprenol carrier, initiating formation of the O-unit in O-antigen assembly. In *L*. *borgpeterseni* serovar Hardjobovis, this protein, encoded by *orfH13*, is an UND-pp-galactosyltransferase [118].

The other major pathway of O-antigen polysaccharide biosynthesis is the *Wzm/Wzt*–encoded or ABC-transporter dependent pathway [119, 120]. Six of the 20 representative genomes encoded orthologs of *Wzm* and *Wzt*. Two of the genomes were from intermediate and four were from saprophytic groups, representing 5 known serovars (e.g. Hurstbridge, Saopaulo, Hualin, Hardjo type Went, and Patoc). The genome analysis did not provide a clear indication of the export system used by serovar Khorat. One other known O-antigen biosynthesis pathway the synthase-dependent pathway [119]. BLASTP searches of WbbE and WbbF from the only known example of this pathway, from the plasmid-encoded O:54 antigen of *S*. *enterica* serovar Borreze [121], failed to identify any homologs in the representative 20 *Leptospira* genomes. It is possible that serovar Khorat uses a novel mechanism for O -antigen biosynthesis.

A dTDP-rhamnose biosynthesis gene cluster, encoding *rfbABCD* was found in the conserved 3-prime end of the predicted O-antigen biosynthetic gene clusters of only pathogenic *Leptospira* spp. serovar Saopaulo, found in the saprophytic species *L*. *yanagawae*, encoded homologs of all four of these genes, but in the order *rfbCBAD*, where the genes *rfbABC* appear to have been inverted (Fig 5C). The genes *rfbAB* and *rfbC*, were found in a different location with *rfbC* separated by several genes in serovars Hualin, Hardjo, and Patoc. These same isolates lacked an *rfbD* homolog. Only *rfbAB* homologs were identified in serovar Hurstbridge.

#### *L*. *licerasiae*-type surface polysaccharide cassettes

We previously reported that *L*. *licerasiae*, which is antigenically unique, lacks the type of extremely large O-antigen biosynthesis region found in *L*. *interrogans* and nearly all other *Leptospira* [122]. Instead, the one serovar of *L*. *licerasiae*, Varillal, has a six-gene cluster with three glycosyltransferase genes between two normally adjacent, convergently transcribed genes: the *murC* gene involved in cell wall biosynthesis and *purK* gene of purine biosynthesis. *Leptospira wolffii* had a similar genomic *rfb* locus, again with a six-gene cluster positioned between *murC* and *purK* (Fig 5B); antigenic relatedness to *L*. *licerasiae* serovar Varillal remains to be confirmed experimentally. The first glycosyltransferase in this cassette, LEP1GSC185_2122 (GenBank EIE02925) in *L*. *licerasiae* and LEP1GSC061_3728 (GenBank EPG64090) in *L*. *wolffii*, are highly conserved and would be a useful marker for this extremely small O-antigen gene cluster. No other protein in the replacement six-gene cassette is conserved across the different variants. Genes in these regions have no close homologs in any other *Leptospira*, in the O-antigen region or anywhere else, supporting the notion that these cassettes provide unique carbohydrate chemistry and serology, and is not simply an unusual gene neighborhood for otherwise common leptospiral enzymes.

#### Lipid A biosynthesis

The lipid A of leptospiral LPS is not as potent an endotoxin as lipid A moieties of other bacteria such as the Enterobacteriaceae or *Neisseria* spp.; the mechanistic explanation for this observation is that *L*. *interrogans* lipid A has different acyl chains and novel phosphorylation on the position of the lipid A that abrogate endotoxinogenicity [123]. The lipid A biosynthetic pathway of *L*. *interrogans* serovar Lai involves 13 enzymes, encoded by genes *lpx*A, *lpx*C, *lpx*D1, *lpx*D2, *lpx*B1, *lpx*B2, *lpx*K, *kdt*A, *kds*B1, *kds*B2, *lnt*, *kds*A (also found as *waa*A) and *htr*B. The presence and homology of amino acid (aa) sequences of these enzymes was compared between 21 different species and/or serovars of *Leptospira* spp classified in three different groups: pathogenic (PT, 10 species), intermediate (IM, 5 species) and non-pathogenic or commensal (NP, 6 species). Most proteins were found in all *Leptospira* species (S6 Table). However *lpx*B2, was found only in 4 pathogenic species/serovar and 1 non-pathogenic, *lpx*D2 was not found in intermediate species/serovar and *htr*B was only present in 1 pathogenic and 1 non-pathogenic species/serovar. The *kds*B1 and *kds*B2 were only found in two species/serovar (*L*. *interrogans* sv. Lai and *L*. *inadai* sv. Lyme), all other species/serovar had only one *kds*B that showed a higher level of similarity with *kds*B2 from *L*. *interrogans* sv. Lai than with *kds*B1. Although we found that some genomes lack one or two lipid A biosynthetic genes (e.g. *lpxD2* and *kdsB2*), the computation analysis is still consistent with functional biosynthetic pathways still being present in all species, because, for the genomes lacking one of the duplicate genes, the remaining ones (e. g. lpxD1 and kdsB1) may be able to complement the function of the lipid A biosynthetic pathway. Another possibility is that the genes are present in the genomes, but we missed the genes because of gene diverge or gaps in genome sequence obtained. Finally, the variable presence of lipid A biosynthesis genes may relate to some as yet undiscovered structural differences in lipid A moieties among *Leptospira*.

The predicted protein sequences of individual lipid A biosynthesis pathway were nearly identical among *Leptospira* as predicted using an identity matrix (S7A Table). The homology between two sequences is expressed within the range of 0 to 1 (identical or completely homologous). The results presented hereby are expressed as the mean of homology values within each group, compared to the pathogenic species group. The *lpx*A amino acid sequence was found in all species, although the average similarity within pathogens was 0.928, while the homology of intermediates and saprophytes was 0.694 and 0.581, respectively, when compared to pathogen sequences. This analysis was carried out for each amino acid sequence (S7B Table).

#### Sialic acids as post-translational modifications restricted to pathogenic *Leptospira*

Previous studies have demonstrated that pathogenic *Leptospira* endogenously synthesize Neu5Ac, the most common sialic acid, and that an observed gene fusion event suggested that *L*. *interrogans* uses a Neu5Ac biosynthetic pathway that is more similar to that of animals than to other bacteria. Lectin-based affinity purification of NulO-modified molecules, followed by mass spectrometric identification suggested post-translational modification of surface lipoproteins, including the putative virulence factor Loa22 [124, 125]. In the genomes analyzed for this study, 3 of the 9 pathogens had the complete cluster of genes involved in the production of sialic acids; 3 more lacked 1 gene in the cluster (Table 3). *L*. *weilii* contains only 2 genes from the cluster (*spsE* and *rfbB3*) and *L*. *kirschneri* and *L*. *noguchii* have only the *spsE* gene

**Table 3:** Leptospiral Proteins for Sialic Acid Biosynthesis.

			*L*. *interrogans* sv. Copenhageni str. Fiocruz L1-130	*L*. *kirschneri* sv. Cynopteri str. 3522 CT	*L*. *noguchii* sv. Panama str. CZ 214T	*L*. *alstoni* sv. Pingchang str. 80–412	*L*. *weilii* sv. undetermined str. LNT 1234	*L*. *alexanderi* sv. Manhao str. 3 L 60T	*L*. *borgpetersenii* sv. Javanica str. UI 09931	*L*. *santarosai* sv. Shermani str. 1342KT	*L*. *kmetyi* sv. Malaysia str. Bejo-Iso9T	*L*. *fainei* sv. Hurstbridge str. BUT 6T	*L*. *broomii* sv. Hurstbridge str. 5399T	*L*. *wolffii* sv. Khorat str. Khorat-H2T	*L*. *licerasiae* sv. Varillal str. VAR 010	*L*. *inadai* sv. Lyme str. 10T	*L*. *wolbachii* sv. Codice str. CDC	*L*. *yanagawae* sv. Saopaulo str. Sao Paulo	*L*. *biflexa* sv. Patoc str. Patoc1	*L*. *vanthielii* sv. Holland str. Waz Holland	*L*. *terpstrae* sv. Hualin str. LT 11-33T	*L*. *meyeri* sv. Hardjo str. Went 5
Protein name	GenBank Accession	Function	P	P	P	P	P	P	P	P	P	I	I	I	I	I	S	S	S	S	S	S
LA_1605	NP_711786.1	CMP-N-acetylneuraminic acid synthetase (*neuA1*)	+	-	-	+	-	+	+	+	+	-	-	-	-	-	-	-	-	-	-	-
LA_1606	NP_711787.1	NAD dependent epimerase/dehydratase family protein (*rfbB3*)	+	-	-	+	+	T	T	+	+	-	-	-	-	-	-	-	-	-	-	-
LA_1607	NP_711788.1	pyridoxal phosphate-dependent aminotransferase	+	-	-	+	-	+	+	+	+	-	-	-	-	-	-	-	-	-	-	-
LA_1608	NP_711789.2	sugar O-acyltransferase, sialic acid O-acetyltransferase NeuD family (*neuD*)	+	-	-	+	-	-	+	+	+	-	-	-	-	-	-	-	-	-	-	-
LA_1609	NP_711790.1	pseudaminic acid synthetase (*neuB*)	+	+	+	+	+	+	+	+	+	+	+	-	-	+	+	+	+	+	+	+
LA_1610	NP_711791.1	UDP-N-acetylglucosamine 2-epimerase (*neuC*)	+	-	-	+	-	+	+	+	+	-	-	-	-	-	-	-	-	-	-	-
LA_1611	NP_711792.1	nucleotidyl transferase	+	-	-	+	-	+	+	+	+	-	-	-	-	-	-	-	-	-	-	-
LA_1612	NP_711793.1	hypothetical protein	+	-	-	+	-	+	+	+	+	-	-	-	-	-	-	-	-	-	-	-
LA_1613	NP_711794.1	N-acetylneuraminic (sialic) acid synthetase (*sas*/*neuB2*)	+	-	-	+	-	+	+	+	+	-	-	-	-	-	-	-	-	+	-	-
LA_1614	NP_711795.1	pyridoxal phosphate-dependent aminotransferase	+	-	-	+	-	+	+	-	-	-	-	-	-	-	-	-	-	+	-	-
LA_1615	NP_711796.1	CMP-N-acetylneuraminic acid synthetase (*neuA2*)	+	-	-	+	-	+	+	+	+	-	-	-	-	-	+	-	-	+	-	-
LA_1645	NP_711826.1	UDP-N-acetylglucosamine 2-epimerase	+	+	+	+	-	+	+	+	+	-	-	-	-	+	+	-	-	+	-	-
LA_3823	NP_714003.1	UDP-N-acetylglucosamine diphosphorylase	+	+	+	+	+	+	+	+	+	+	+	+	+	+	+	+	+	+	+	+

P = Pathogenic species; I = Intermediate species; S = Saprophytic species

T = Truncation

All genomes, except *L*. *licerasiae* and *L*. *wolffii* have a N-acetylneuraminic (sialic) acid synthetase (*spsE*) gene (NP_711790.1). Phylogenetic analysis of this protein shows 2 distinct groups (S3 Fig). The first group contains the proteins from pathogens that contain the whole cluster. These proteins are related to the synthetases involved in the production of legionaminic acids. The second group contains the proteins from the intermediate species, the saprophytes and the pathogens *L*. *kirschneri* and *L*. *noguchii*. This group of synthetases is related to those producing pseudaminic acids.

The lack of a second sialic acid synthetase (NP_711794.1) in *L*. *kirschneri* and *L*. *noguchii* differentiates these pathogens from *L*. *interrogans*, which does contain this gene. These synthetases contain a phosphatase domain in addition to the NeuB domain, which suggests an animal-like Neu5Ac biosynthetic pathway. The pathogen *L*. *weilli* lacks NP_711794.1 but saprophyte *L*. *vanthielli* contains a similar synthetase but one that is missing the N-terminal transferase domain present in leptospiral pathogens. Finally, a UDP-N-acetylglucosamine diphosphorylase (NP_714003.1) was found in all leptospiral genomes studied. This gene is not located within the sialic acid gene cluster, and is also annotated as a MobA-like NTP transferase domain, therefore its role in sialic acid biosynthesis is unclear.

The sialic acid biosynthetic genes in leptospiral pathogens have some notable characteristics. *L*. *alexanderi* lacks O-acyltransferase (*neuD*) and this species and *L*. *borgpetersenii* have a truncated version of a nucleoside-diphosphate-sugar epimerase (NP_711787.1) (S3 Fig; Table 3). Only *L*. *santarosai* has a N-acetylneuraminic (sialic) acid synthetase with a phosphoglycerate dehydrogenase domain. Notably, none of the intermediate or saprophyte species contain the metabolic machinery to synthesize sialic acids, confirming previous suggestions [124].

### Leptospiral mobile elements: phage and CRISPR-Cas systems

#### Phage

Bacteriophages are abundant biological entities that have significant effects on bacterial evolution. Some estimates suggest that there are approximately ten-fold more phages than bacteria [126, 127]. However, our current knowledge of phages infecting *Leptospira* spp. is limited. Three distinct *L*. *biflexa* phages were isolated from sewage water in Paris. Morphological analysis by electron microscopy revealed that these three phages belong to the *Myoviridae* family and seem to be morphologically similar with polyhedral heads and contractile tails [128]. One of these phages, the 74-kb LE1 prophage, was shown to replicate as a double-stranded circular replicon in *L*. *biflexa* [129]. The genome of LE1 has a GC content of 36%, similar to that of *Leptospira* spp., and most of the 79 predicted ORFs display no similarity to known ORFs, but 21 ORFs appeared to be organized in clusters that might encode head and tail structural proteins and immunity repressor proteins [130].

Next generation sequencing and refinement of computational methods have allowed comparative genome analysis to discover new prophage and genomic islands [131]. A few phage related genomic islands have thus been characterized in *L*. *interrogans* and *L*. *licerasiae* [122, 132, 133] and it was previously shown that one of these genomic islands can excise from the *L*. *interrogans* chromosome [133].

To determine the distribution of prophages within the *Leptospira* genus, Phage_Finder [60] was run under both strict (-S) and non-strict modes to identify predicted prophage regions. Phage_Finder predicted a total of 14 major prophage regions across the 20 genomes, most of which were found to be shared between the *Leptospira* species (Table 4). Among the prophage sequences, the LE1-like prophage is found in many genomes, suggesting that double-stranded DNA tailed phages, which are the most frequently observed phages in bacteria [134], are common phages infecting *Leptospira*. The presence of numerous phage-associated sequences in the genome of pathogens and intermediates, in comparison to the saprophytes, suggests that phages have played an important part in the evolution of these lineages, as has been experimentally shown in *L*. *biflexa* [135].

**Table 4:** Predicted *Leptospira* Prophage Regions.

					Contig	Region					
	Species	Name*	Topology	Accession	Size (bp)	Size (bp)	GC%	Begin	End	Best Non-self DB Match (#/total with match)	Family
**pathogenic**	*alexanderi*	vB_LalZ_L60-AHMT02000036_PFPR01	linear	AHMT02000036	95771	17843	42.80	LEP1GSC062_0091	LEP1GSC062_0117	*L*. *licerasiae* prophage vB_LliZ_VAR010_Qinetal (22/23)	Qinetal-like
		vB_LalZ_L60-LE1	linear	AHMT02000050	47777	42754	41.67	LEP1GSC062_4268	LEP1GSC062_4321	*L*. *licerasiae* circular prophage vB-LliZ_VAR10-LE1 (39/42)	LE1-like
	*alstoni*	vB_LalZ_80412-LE1	linear	KF114876	86537	54740	39.08	LEP1GSC193_0706	LEP1GSC193_0771	*L*. *licerasiae* circular prophage vB-LliZ_VAR10-LE1 (40/47)	LE1-like
		vB_LalZ_80412-AOHD02000025_PFPR01	linear	AOHD02000025	333520	35036	42.48	LEP1GSC193_2291	LEP1GSC193_2345	*L*. *licerasiae* prophage vB_LliZ_VAR010_Qinetal (42/43)	Qinetal-like
	*borgpetersenii*	No phages were detected in this genome									
	*interrogans*	No phages were detected in this genome									
	*kirschneri*	No phages were detected in this genome									
	*kmetyi*	vB_LkmZ_BejoIso9-LE1	circluar	AHMP02000002	79000	60209	39.48	LEP1GSC052_0001	LEP1GSC052_0055	*L*. *licerasiae* circular prophage vB-LliZ_VAR10-LE1 (35/38)	LE1-like
	*noguchii*	vB_LnoZ_CZ214-AKWY02000031_PFPR02	linear	AKWY02000031	180862	40313	41.34	LEP1GSC059_0671	LEP1GSC059_0731	*Listeria* phage B054 (7/18)	Mu-like?
		vB_LnoZ_CZ214-AKWY02000034_PFPR02	linear	AKWY02000034	690650	32483	42.42	LEP1GSC059_2330	LEP1GSC059_2380	*Listeria* phage B054 (7/15)	Mu-like?
		vB_LnoZ_CZ214-AKWY02000016_PFPR01	linear	AKWY02000016	106299	32482	42.42	LEP1GSC059_3177	LEP1GSC059_3225	*Listeria* phage B054 (7/14)	Mu-like?
		vB_LnoZ_CZ214-AKWY02000018_PFPR01	linear	AKWY02000018	118954	32484	42.41	LEP1GSC059_3647	LEP1GSC059_3696	*Listeria* phage B054 (7/14)	Mu-like?
		vB_LnoZ_CZ214-LE1	circular	KF114877	87887	87887	33.96	LEP1GSC059_0001	LEP1GSC059_0097	*L*. *licerasiae* circular prophage vB-LliZ_VAR10-LE1 (39/50)	LE1-like
	*santarosai*	vB_LsaZ_1342K-AOHB02000057_PFPR01	linear	AOHB02000057	174636	36190	39.10	LEP1GSC048_3923	LEP1GSC048_3972	*L*. *licerasiae* prophage vB_LliZ_VAR010_Qinetal (10/23)	Qinetal-like
		vB_LsaZ_1342K-AOHB02000043_PFPR01	linear	AOHB02000043	157378	26431	41.19	LEP1GSC048_0650	LEP1GSC048_0689	*L*. *licerasiae* prophage vB_LliZ_VAR010_Qinetal (12/23)	Qinetal-like
		vB_LsaZ_1342K-AOHB02000048_PFPR01	linear	AOHB02000048	94407	38273	43.44	LEP1GSC048_2654	LEP1GSC048_2709	*L*. *interrogans* prophage vB_LinZ_Lai_Qinetal (22/35)	Qinetal-like
	*weilii*	vB_LweZ_LNT1234-AHNC02000072_PFPR01	linear	AHNC02000072	12189	11900	44.91	LEP1GSC086_0007	LEP1GSC086_0022	*L*. *licerasiae* prophage vB_LliZ_VAR010_Qinetal (16/16)	Qinetal-like
		vB_LweZ_LNT1234-AHNC02000035_PFPR01	linear	AHNC02000035	118346	36760	42.30	LEP1GSC086_4186	LEP1GSC086_4242	L. interrogans prophage vB_LinZ_Lai_Qinetal (22/36)	Qinetal-like
**intermediate**	*broomii*	vB_LbrZ_5399-LE1	linear	KF114879	87308	43991	39.40	LEP1GSC050_0008	LEP1GSC050_0058	*L*. *licerasiae* circular prophage vB-LliZ_VAR10-LE1 (15/21)	LE1-like
	*fainei*	No phages were detected in this genome									
	*inadai*	vB_LinZ_10-LE1	circular	KF114880	89607	62135	39.87	LEP1GSC047_0831	LEP1GSC047_0905	*L*. *licerasiae* circular prophage vB-LliZ_VAR10-LE1 (37/41)	LE1-like
	*licerasiae*	vB_LliZ_VAR010-LE1	circular	AHOO02000007	103186	103186	37.80	LEP1GSC185_3903	LEP1GSC185_3949	*L*. *biflexa* temperate bacteriophage LE1 (21/28)	LE1-like
		vB_LliZ_VAR010-AHOO02000005_PFPR01	linear	AHOO02000005	1668761	38621	42.84	LEP1GSC185_097	LEP1GSC185_093	*L*. *licerasiae* prophage vB_LliZ_VAR010_Qinetal (12/23)	Qinetal-like
	*wolffii*	No phages were detected in this genome									
**saprophytic**	*biflexa*	No phages were detected in this genome									
	*meyeri*	No phages were detected in this genome									
	*terpstrae*	No phages were detected in this genome									
	*vanthielii*	No phages were detected in this genome									
	*wolbachii*	No phages were detected in this genome									
	*yanagawae*	No phages were detected in this genome									

*Systematic names were only given to prophages matching sequences of known bacteriophages that are present in one copy in the genome. All other names are a combination of systematic nomenclature plus the GenBank accession of the contig the prophage was identified on followed by the Phage_Finder.pl id (i.e., PRPR#).

Further experimental studies of *Leptospira* phages, which would include both electron microscopic visualization and production of phage *in vitro*, will be important to determine whether recombinant *Leptospira* phage might be useful for genetic manipulation studies of different *Leptospira* species, particularly pathogens and intermediates.

#### CRISPR/Cas systems

Three described types of CRISPR/Cas systems are common in *Leptospira* genomes and only found in infectious members of the genus (Table 5): the *E*. *coli* (type I-E), DVULG (type I-C), and MYXAN systems [96, 136, 137]. A single sequenced genome, *L*. *inadai* serovar Lyme str. 10, has the recently described PreFran type, which has been found in *Prevotella* and *Francisella* [96]. Four of the 20 representative strains have components of two CRISPR/Cas types, suggesting that for some isolates there is redundancy in CRISPR/Cas machinery. Surprisingly, half of the 20 representative *Leptospira* strains contained predicted CRISPR repeats, which were more common in pathogens and intermediates than in saprophytes, which lacked CRISPR systems. In none of the six saprophytes examined—*L*. *biflexa*, *L*. *meyeri*, *L*. *wolbachii*, *L*. *vanthielii*, *L*. *yanagawae*, and *L*. *terpstrae*—were CRISPR/Cas systems detected, suggesting that these species rely on some other mechanism for escaping phage/plasmid attack. In these saprophytes, we were also unable to detect sequences encoding prophage, while CRISPR systems and prophage occurred together in several, but not all, representative pathogenic and intermediate strains.

**Table 5:** CRISPR/Cas Systems Identified in *Leptospira* Species Representatives.

		CRISPR Arrays†				CRISPR System Genome Properties Evidence*			
Species	Lifestyle	Contig	Direct Repeat Consensus	# Spacers	Location	Dvulg (type I-C)	Ecoli (type I-E)	Myxan	PreFran
*alexanderi*	pathogenic	AHMT02000065	CGGTTCAACCCCACGCATGTGGAGAATAG	16	6749..7777	0	1	0	0
		AHMT02000052	CGGTTCAACCCCACGTGTGTGGGGAAAAG	25	65616..67218				
		AHMT02000009	……AACCCCACGTGTGTGGGGAAAAG	6	11327..11714				
*alstoni*	pathogenic	AOHD02000029	GTGCTCAACGCCTAACGGCATCAAAGGTATGTTCAG	14	329..1366	0	0.125	0.444	0
*borgpetersenii*	pathogenic	NA	NA	NA	NA	0	0.875	0	0
*interrogans*	pathogenic	AE016823.1	GTGCTCAACGCCTAACGGCATCAAAGTTATATTCAG	3	1133848..1134101	1	0	0.889	0
		AE016823.1	TTCCTAAAGAAATAGGGAATTTAAAAAA	4	1451041..1451345				
*kirschneri*	pathogenic	AHMN02000020	GTGCTCAACGCCTTACGGCATCAAAGTTAAGTTCAG	5	13791..14191	1	0	0.556	0
		AHMN02000010	ATTTCTTTCTCTTATTAAAGAGGAAGTGGATTGAAAC	3	88470..88720				
*kmetyi*	pathogenic	NA	NA	NA	NA	0	0	0	0
*noguchii*	pathogenic	AKWY02000022	GTTTCAATCCACTCTTCTCTAAAAGAAGAGATTAAAC	12	56305..57198	1	0	0	0
		AKWY02000014	..TTCAATCCACTTCCTTCACAAACAAAGGAAGAAAT	5	7603..7996				
		AKWY02000003	GTTTCTTTCTCTTGTTTGAGAGGAAGTGGATTGAAAC	10	55925..56687				
		AKWY02000021	GTTTCAATCCACTTCCTTTTTAAACCAAGGAAGAAAC	7	48415..48952				
		AKWY02000021	GTTTCTTCTTCTTACTTTAGAGGAAGTGGATTGAAAC	8	315674..316283				
		AKWY02000021	GTTTCAATCCGCTTCCTCTTTAATAAGAGAAAGAAAT	4	423849..424171				
*santarosai*	pathogenic	AOHB02000026	CGGTTCAACCCCACGCATGTGGGGAAAAG	5	19990..20321	0	1	0.889	0
		AOHB02000050	CGGTTCAACCCCACGCATGTGGGGAAAAG	9	16484..17061				
		AOHB02000050	GTGCTCAACGCCTTACGGCATCAAAGTTATATTCAG	3	50240..50492				
		AOHB02000050	.TGCTCAACGCCTTACGGCATCAAAGTTATATTCAG	3	52981..53232				
		AOHB02000003	CTTTTCCCCACATGCGTGGGGTTGAACCG	3	56764..56975				
*weilii*	pathogenic	AHNC02000050	CTTTTCCCCACATGCGTGGGGTTGAACCG	7	80411..80865	0	0.875	0	0
		AHNC02000082	CTTTTCCCCACACACGTGGGGTTGAACCG	16	222247..223251				
*broomii*	intermediate	AHMO02000008	CTGAAACTAACTTTGATGCCGAAAGGCGTTGAGCAT	11	908793..909615	0	0	1	0
*fainei*	intermediate	AKWZ02000010	CGGTTCATCCCCACGTGTGTGGGGAATAG	5	1694300..1694633	0	1	0	0
		AKWZ02000010	CGGTTCATCCCCACGTGTGTGGGGAATAG	6	1695946..1696339				
		AKWZ02000004	CTATTCCCCACACACGTGGGGATGAACCG	18	200759..201886				
*inadai*	intermediate	AHMM02000017	GTTTCAATTCCAGATTGGTTCGATTAAAAG	22	566571..568061	0	0	0	1
		AHMM02000017	ATCTACAAAAGTAGAAATTCTTTCCTCTCTTTAGAG	9	625117..625713				
*licerasiae*	intermediate	NA	NA	NA	NA	0	0	0	0
*wolffii*	intermediate	NA	NA	NA	NA	0	0	0	0
*biflexa*	saprophytic	NA	NA	NA	NA	0	0	0	0
*meyeri*	saprophytic	NA	NA	NA	NA	0	0	0	0
*terpstrae*	saprophytic	NA	NA	NA	NA	0	0	0	0
*vanthielii*	saprophytic	NA	NA	NA	NA	0	0	0	0
*wolbachii*	saprophytic	NA	NA	NA	NA	0	0	0	0
*yanagawae*	saprophytic	NA	NA	NA	NA	0	0	0	0

^†^Predicted using CRISPRFinder (Grissa et al., 2007).

*Evidence value ranges from 0 (absent) to 1 (present; all steps of the genome property were found)

NA = Not applicable because no "confirmed" CRISPR repeats were identified

**Fig 6:**
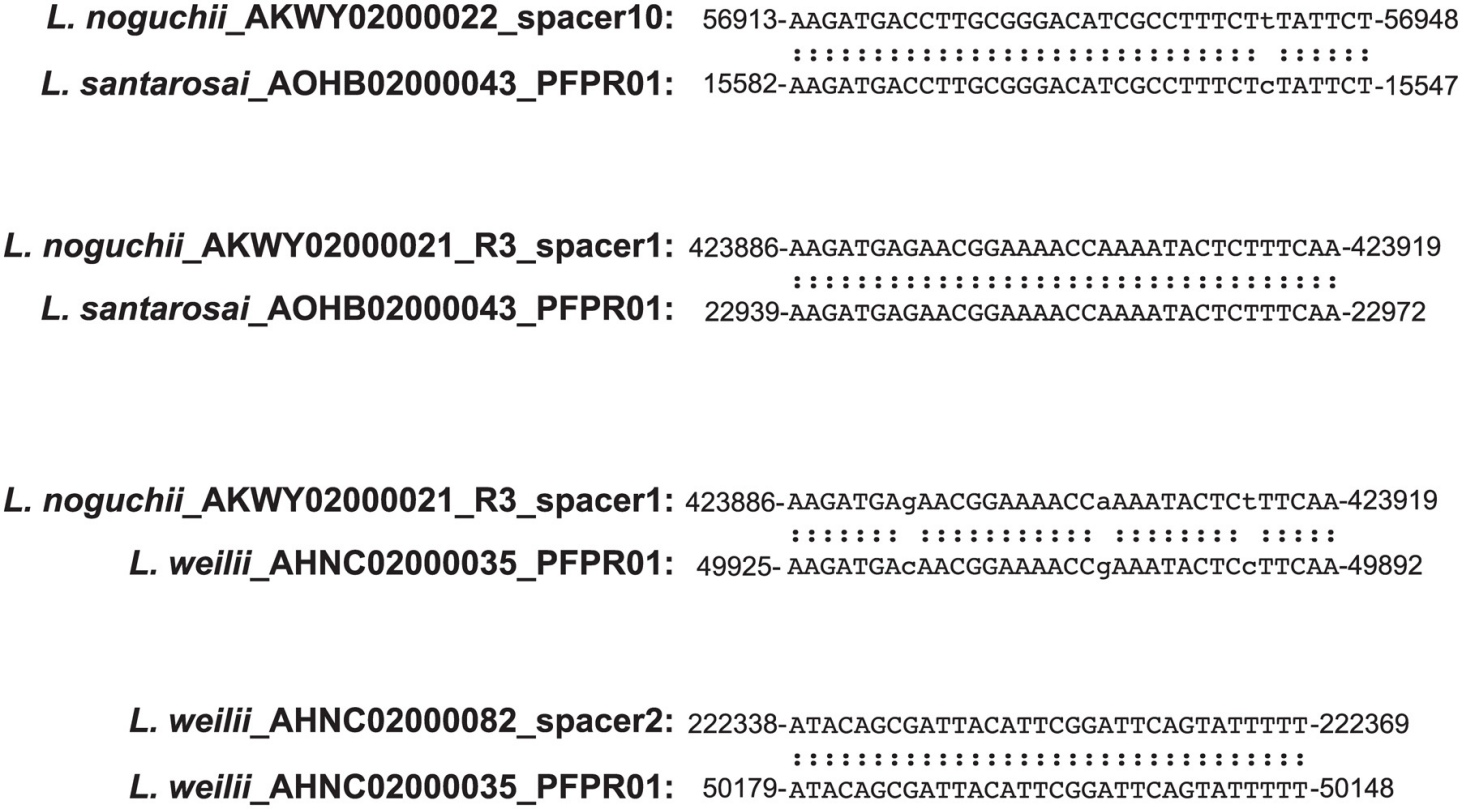
CRISPR Spacer Sequences that Recognize *Leptospira* Predicted Prophages. The CRISPR sequences are shown, which correspond to specific prophage accession numbers as listed in Table 4.

When present, between one and six CRISPR repeat arrays were detected, containing between three and 25 spacer sequences (Table 5). Since CRISPR spacer sequences in other organisms are known to target phage sequences for destruction, we wondered if any of the 239 predicted spacer sequences targeted any of the known *Leptospira* spp. phage or predicted prophages. A database containing the nucleotide sequences of the 19 predicted prophages from this study plus the LE1 phage and the prophage from Qin et al. [32, 132] was constructed and used to search all 239 predicted spacer sequences using BLAST+ 2.2.30 [138]. Upon filtering the data for matches spanning the entire spacer sequence, with 3 or fewer mismatches and with a bitscore of 30+ revealed three spacer sequences matching two predicted prophage sequences (Fig 6). Two different *L*. *noguchii* spacers matched the same predicted *L*. *santarosai* prophage (Table 5). One of the same *L*. *noguchii* spacers also matched a predicted *L*. *weilii* prophage and was also recognized by an *L*. *weilii* spacer (Fig 6).

### Virulence and Survival Mechanisms

#### Adhesion to Extracellular Matrix (ECM)

The presence of genes encoding putative adhesive proteins through the 20 sequenced species was analyzed by BLAST and comparative genome analysis (S8 Table). The widespread distribution of these genes within the *Leptospira* genus suggests that their functions arose independent of mammalian adaptation, but any potential role in adaptation to an environmental lifestyle remains speculative. These predicted adhesion-related proteins were generally distributed among the 20 species except for the predicted adhesin-encoding gene *LenB*, identified only in pathogenic *L*. *interrogans* serovars Lai and Copenhageni strains; in the saprophyte *L*. *meyeri*; and Lsa27, Lsa21, LipL53 present in two, three and five pathogen species, respectively. Three predicted adhesin-encoding genes were restricted to pathogenic species: *Lsa30*, *Lsa44* and *Mfn6*. *Lsa30* was present in all species, *Lsa44* was absent in *L*. *interrogans* serovar Lai and *L*. *weilli*, and *Mfn6* was absent in *L*. *weilli* and *L*. *alexanderi*. The genes encoding *Lsa23*, *Lsa26*, *Lsa33*, *Lsa45*, *Lsa66*, *LipL32* and *Mfn1* were found in all infectious species (pathogens and intermediates) but absent in saprophytes. The Len protein family members were variably distributed. *Lsa24/LenA* was identified in all sequenced strains, while *LenB*, *LenC*, *LenD*, *LenE* and *LenF* were found in both pathogens and saprophytes; *LenD* was also found in the intermediate *L*. *wolffii*. *Lsa23*, *Lsa26*, *Lsa33*, *Lsa45*, *Lsa66*, *LipL32* and *Mfn1* were found in all pathogenic and intermediate but are absent in saprophyte strains. The Len protein family showed a random distribution among the genome species: *Lsa24/LenA* was identified in all sequenced strains, while *LenB*, *LenC*, *LenD*, *LenE* and *LenF* were found in both pathogenic and saprophytic *Leptospira*, and *LenD* was also found in *L*. *wolffii*, an intermediate. The adhesins *Lsa20*, *Lsa25*, *Lsa36*, *Lsa63*, *TlyC*, *OmpL1*, *OmpL37*, *OmpL47*, *Mfn7* and *rLIC12976* were identified in all pathogenic, intermediate and saprophyte species; *Mfn9* was found in all except in *L*. *santarosai*. Also listed are plasminogen- and complement regulator-binding proteins (S8 Table) that have functions related to the predicted proteins listed above.

#### Complement evasion and ECM degradation via metalloproteases

Two leptospiral proteases have been suggested as virulence factors: thermolysin and collagenase. Thermolysins are members of the M4 metalloprotease family that can be identified bioinformatically by the presence of two N-terminal propeptide (FTP, and PepSY) and two C-terminal protease domains (Peptidase_M4 and Peptidase_M4_C) [139]. Using Pfam HMMs targeting these four domains (e.g., PF07504, PF03413, PF012868 and PF01447), we identified *LIC13322* and four additional predicted thermolysin orthologs only in pathogenic *Leptospira* spp.: *LIC10715*, *LIC13320*, *LIC13321*, and *LEP1GSC059_0182* (S9 Table), primarily among *L*. *interrogans*, *L*. *kirschneri* and *L*. *noguchii*. LEP1GSC059_0182 was found only in one species, *L*. *noguchii*. No thermolysin ortholog was found in intermediate or saprophytic species.

Collagenase has been suggested to be a virulence factor in *Leptospira* based on observed *in vivo* expression, detection of specific anti-collagenase antibodies induced by infection, and the effects of *ColA* mutagenesis and complementation on traversal of cell monolayers and outcome of experimental animal infection [140, 141]. Comparative genomic analysis identified two collagenase genes, restricted to pathogenic *Leptospira* spp.: orthologs of one (LIC_12760) were found in all pathogens except *L*. *kmetyi*; an additional paralog, EMN46521, was restricted to *L*. *weilii* and *L*. *alexanderi*, and based on nearly identical size and closely related amino acid sequences, likely arose by gene duplication (S9 Table). The implications of this latter finding for pathogenesis are unclear.

#### Resistance to oxidative stress

Three enzyme systems have conventionally been associated with the ability of pathogenic bacteria to defend against host-derived oxidative stress-related mediators such as hydrogen peroxide and superoxide radicals: catalases, peroxidases and superoxide dismutase. While catalases generate water and oxygen from H_2_0_2_, peroxidases generate water and an oxygen radical.

*KatA* and another but uncharacterized predicted catalase ortholog (LEP1GSC062_4039) were only found in pathogenic *Leptospira*, suggesting that these enzymes play an important role for *Leptospira* living within the mammalian host (Table 6). Conversely, superoxide dismutase was not found in pathogenic *Leptospira* but only in saprophytic *Leptospira*, suggesting either that pathogenic *Leptospira* are not exposed to oxygen radicals in the environment, or, more likely, that this clade of *Leptospira* has developed alternative ways to detoxify oxygen radicals.

**Table 6:** Proteins involved in the resistance to oxidative stress.

			*L*. *interrogans* sv. Copenhageni str. Fiocruz L1-130	*L*. *kirschneri* sv. Cynopteri str. 3522 CT	*L*. *noguchii* sv. Panama str. CZ 214T	*L*. *alstoni* sv. Pingchang str. 80–412	*L*. *weilii* sv. undetermined str. LNT 1234	*L*. *alexanderi* sv. Manhao str. 3 L 60T	*L*. *borgpetersenii* sv. Javanica str. UI 09931	*L*. *santarosai* sv. Shermani str. 1342KT	*L*. *kmetyi* sv. Malaysia str. Bejo-Iso9T	*L*. *fainei* sv. Hurstbridge str. BUT 6T	*L*. *broomii* sv. Hurstbridge str. 5399T	*L*. *wolffii* sv. Khorat str. Khorat-H2T	*L*. *licerasiae* sv. Varillal str. VAR 010	*L*. *inadai* sv. Lyme str. 10T	*L*. *wolbachii* sv. Codice str. CDC	*L*. *yanagawae* sv. Saopaulo str. Sao Paulo	*L*. *biflexa* sv. Patoc str. Patoc1	*L*. *vanthielii* sv. Holland str. Waz Holland	*L*. *terpstrae* sv. Hualin str. LT 11-33T	*L*. *meyeri* sv. Hardjo str. Went 5
Cluster Representative	GenBank Accession	Function	P	P	P	P	P	P	P	P	P	I	I	I	I	I	S	S	S	S	S	S
LEP1GSC062_0898	EQA63414	catalase (*KatA*)	+	+	+	+	+	+	+	+	+	−	−	+	−	−	−	−	−	−	−	−
LEP1GSC195_0257	EOQ97809	catalase/peroxidase (*KatG*)	−	−	−	−	−	−	−	−	−	−	−	−	−	−	+	+	+	+	+	+
LEP1GSC062_4039	EQA64708	catalase	+	+	+	+	+	+	+	+	+	−	−	−	+	−	−	−	−	−	−	−
LEP1GSC086_1809	EMN42918	di-heme cytochrome C peroxidase	+	+	+	+	+	+	+	−	+	−	−	+	−	−	+	−	+	+	+	+
LEP1GSC086_3491	EMN44421	di-heme cytochrome C peroxidase	+	+	+	+	+	+	+	+	+	+	+	+	+	+	−	−	−	−	−	−
LEP1GSC052_4289	EQA52716	di-heme cytochrome C peroxidase	+	+	+	+	+	+	+	+	+	+	+	+	+	+	+	+	+	+	+	+
LEP1GSC203_3912	EMY61934	di-heme cytochrome C peroxidase	−	−	−	−	−	−	−	−	−	−	−	−	−	−	+	+	+	+	+	+
LEP1GSC062_2886	EQA62707	glutathione peroxidase	+	+	+	+	+	+	+	+	+	+	+	+	+	+	+	+	+	+	+	+
LEP1GSC052_4239	EQA53474	glutathione peroxidase	+	+	+	+	+	+	+	+	+	+	+	+	+	−	+	+	+	+	+	+
LEP1GSC059_3768	EQA72367	glutathione peroxidase	+	+	+	+	+	+	+	+	+	+	+	+	+	+	+	+	+	+	+	+
LEP1GSC195_3278	EOQ97317	superoxide dismutase	−	−	−	−	−	−	−	−	−	−	−	−	−	−	+	+	+	+	+	+

P = Pathogenic species; I = Intermediate species; S = Saprophytic species

#### Immunodominant proteins of *Leptospira*

Previously published protein microarray analysis demonstrated the presence of immunodominant proteins of *L*. *interrogans* serovar Copenhageni using sera from confirmed leptospirosis cases in Bahia state, Brazil. The top 24 immunogenic hits from this analysis were analyzed throughout the genus *Leptospira*, focusing on the presence of orthologs and their amino acid similarities (S10A Table). Only 1 of these genes (a methyltransferase, NC_005823.1) was restricted to *L*. *interrogans*, and only 1 (Lig A) was found in only *L*. *interrogans* and *kirschneri*, with the caveat that a 56% homologous LigA domain was found in *L*. *alstoni*, a leptospire with unclear disease potential. Orthologs of 20 of these 24 hits were detected in all *Leptospira* species but with variable amino acid similarities, suggesting that species- (and perhaps serovar-) specific protein microarrays might be necessary for accurate assessment of immune responses induced by different *Leptospira* in humans. Further, to validate such arrays with well-defined sera from leptospirosis cases, identification of the infecting leptospire will be necessary.

The extensively-studied leptospiral immunoglobulin-like (Lig) protein family is comprised of three proteins, LigA, LigB and LigC [142, 143], which have bacterial immunoglobulin-like (Big) repeat domains, a motif found in virulence factors of other bacterial pathogens [144, 145]. The three genes encoding Lig proteins were believed to be pathogen-specific [142, 146–148]. Comparative analysis of 20 genomes confirmed that *ligA* and *ligB* were present exclusively in pathogenic *Leptospira*: *ligB* was identified in all pathogenic species, *ligA* was found in three of the nine pathogenic species, *L*. *alstoni*, *L*. *kirschneri* and *L*. *interrogans* (S10B Table). While *ligC* was found in the five intermediate species and five of nine pathogenic species (S10B Table), none of the *lig* genes were identified in genomes of saprophytes. *ligC* was previously identified as a pseudogene from sequence analysis of a limited number of strains [142, 147, 148].

The unique structure of LigA, LigB and LigC proteins, which includes a large number of tandem Big domains, is conserved across species for which *lig* genes were found. *ligB* and *ligC* encode molecules which are comprised of a lipobox sequence, 12 tandem Big2 type domains and a C-terminal non-Big domain (S10B Table), whereas *ligA* encodes a protein with 13 tandem Big domains which lacks a C-terminal non-Big domain. Of note, further prospection of the genome sequence and ortholog families identified four additional genes that encode Big2 and Big3_4 domain-type containing proteins (S10B Table). These genes are different from the conventional *lig* gene family in that they encode proteins with a small (1–2) number of Big domains. Interestingly, a gene (LIC13050) encoding a protein with two Big3_4 domains was found in all *Leptospira* species, including the saprophytes.

#### PF07598 paralogous gene family

Previous work identified a group I-specific family of proteins corresponding to Pfam model PF07598 [149] that was expressed *in vivo* in a hamster model of acute leptospirosis [149], and expanded in strains, e.g., Copenhageni and Lai, that commonly cause severe disease, suggesting that these proteins contribute to *Leptospira* virulence. These prior studies focused on finding PF07598 orthologs to the *L*. *interogans* Lai attenuated strain in the 20 representative *Leptospira* spp., but did not look for strain-specific homologs that match PF07598 HMM. To identify novel PF07598 family members, we identified clusters of protein orthologs from our pan-genome run that matched PF07598 above trusted cut-offs; this analysis also included matches within the genomes of previously sequenced strains *L*. *borgpetersenii* Hardjo L550 and JB197 (and previously annotated as conserved hypothetical proteins). At least 26 distinct orthologs ranging in length from 47 (LEP1GSC049_1303 unique to *L*. *kirschneri*) amino acids to 651 (LEP1GSC193_2756; *L*. *alstoni* 80–412) were identified (Table 7; only homologs longer than 200 amino acids shown). As previously reported, *L*. *santarosai* 1342KT contains two distinct homologs, while *L*. *kirschneri* 3522 CT at least 15 and *L*. *noguchii* CZ214 at least 14 including 5 (LEP1GSC059_0232, LEP1GSC059_3018, LEP1GSC059_3019, LEP1GSC059_3599 and LEP1GSC059_3600) without an apparent ortholog in any of the other strains tested (Table 7). *L*. *borgpetersenii* Javanica (4 total) and the previously sequenced genomes of two Hardjo strains (3) contain two orthologs in common, while Javanica has two distinct copies (LEP1GSC103_4030 and LEP1GSC103_0672) not present in Hardjo and both Hardjo strains share an ortholog (LBJ_1339 and LBL_1564) not present in Javanica. In addition, *L*. *interrogans* L1-130 contains an ortholog (LIC_10639) shared with *L*. *noguchii* CZ214T and *L*. *kirschneri* 3522 CT not present in *L*. *interrogans* 56601 consistent with the hypothesis that serovar Lai has lost this ortholog.

**Table 7:** Distribution of the PF07598 paralogous gene family in *Leptospira*.

			*L*. *interrogans* sv. Copenhageni str. Fiocruz L1-130	*L*. *kirschneri* sv. Cynopteri str. 3522 CT	*L*. *noguchii* sv. Panama str. CZ 214T	*L*. *alstoni* sv. Pingchang str. 80–412	*L*. *weilii* sv. Undetermined str. LNT 1234	*L*. *alexanderi* sv. Manhao str. 3 L 60T	*L*. *borgpetersenii* sv. Javanica str. UI 09931	*L*. *santarosai* sv. Shermani str. 1342KT	*L*. *kmetyi* sv. Malaysia str. Bejo-Iso9T	*L*. *fainei* sv. Hurstbridge str. BUT 6T	*L*. *broomii* sv. Hurstbridge str. 5399T	*L*. *wolffii* sv. Khorat str. Khorat-H2T	*L*. *licerasiae* sv. Varillal str. VAR 010	*L*. *inadai* sv. Lyme str. 10T	*L*. *wolbachii* sv. Codice str. CDC	*L*. *yanagawae* sv. Saopaulo str. Sao Paulo	*L*. *biflexa* sv. Patoc str. Patoc1	*L*. *vanthielii* sv. Holland str. Waz Holland	*L*. *terpstrae* sv. Hualin str. LT 11-33T	*L*. *meyeri* sv. Hardjo str. Went 5
Protein name	Genbank Accesion	Function	P	P	P	P	P	P	P	P	P	I	I	I	I	I	S	S	S	S	S	S
LEP1GSC049_0112	EPG49368.1	PF07598 family protein	-	+	+	-	-	-	-	-	-	-	-	-	-	-	-	-	-	-	-	-
LA_3388	NP_713568.1	conserved hypothetical protein	+	+	-	-	-	-	-	-	-	-	-	-	-	-	-	-	-	-	-	-
LA_3271	NP_713451.1	conserved hypothetical protein	+	+	-	-	-	-	-	-	-	-	-	-	-	-	-	-	-	-	-	-
LA_2628	NP_712809.1	conserved hypothetical protein	+	+	-	-	-	-	-	-	-	-	-	-	-	-	-	-	-	-	-	-
LEP1GSC049_1303	EPG49097.1	hypothetical protein	-	+	-	-	-	-	-	-	-	-	-	-	-	-	-	-	-	-	-	-
LEP1GSC049_1343	EPG48826.1	PF07598 family protein	-	+	+	-	-	-	-	-	-	-	-	-	-	-	-	-	-	-	-	-
LEP1GSC049_1381	EPG49151.1	PF07598 family protein	+	+	+	-	-	-	-	-	-	-	-	-	-	-	-	-	-	-	-	-
LA_3490	NP_713670.1	conserved hypothetical protein	+	+	+	+	+	-	-	-	-	-	-	-	-	-	-	-	-	-	-	-
LA_0934	NP_711115.1	conserved hypothetical protein	+	+	-	-	-	-	-	-	-	-	-	-	-	-	-	-	-	-	-	-
LA_1400	NP_711581.2	conserved hypothetical protein	+	+	+	-	-	-	-	-	-	-	-	-	-	-	-	-	-	-	-	-
LEP1GSC049_1750	EPG51109.1	hypothetical protein	-	+	+	-	+	-	-	-	-	-	-	-	-	-	-	-	-	-	-	-
LA_1402	NP_711583.2	conserved hypothetical protein	+	+	+	+	+	+	+	+	-	-	-	-	-	-	-	-	-	-	-	-
LA_0835	NP_711016.1	conserved hypothetical protein	+	+	-	-	-	-	-	+	-	-	-	-	-	-	-	-	-	-	-	-
LA_0769	NP_710950.2	conserved hypothetical protein	+	+	+	-	-	-	-	-	-	-	-	-	-	-	-	-	-	-	-	-
LA_0620	NP_710801.1	conserved hypothetical protein	+	+	+	-	-	-	-	-	-	-	-	-	-	-	-	-	-	-	-	-
LA_0589	NP_710770.1	conserved hypothetical protein	+	+	+	+	-	+	+	-	-	-	-	-	-	-	-	-	-	-	-	-
LEP1GSC049_3371	EPG50678.1	PF07598 family protein	-	+	+	-	-	-	-	-	-	-	-	-	-	-	-	-	-	-	-	-
LA_0591	NP_710772.1	hypothetical protein	+	-	+	+	+	-	+	-	-	-	-	-	-	-	-	-	-	-	-	-
LEP1GSC059_0232	EQA73319.1	PF07598 family protein	-	-	+	-	-	-	-	-	-	-	-	-	-	-	-	-	-	-	-	-
LEP1GSC059_3018	EQA71544.1	PF07598 family protein	-	-	+	-	-	-	-	-	-	-	-	-	-	-	-	-	-	-	-	-
LEP1GSC059_3019	EQA71583.1	hypothetical protein	-	-	+	-	-	-	-	-	-	-	-	-	-	-	-	-	-	-	-	-
LEP1GSC059_3599	EQA70772.1	PF07598 domain protein	-	-	+	-	-	-	-	-	-	-	-	-	-	-	-	-	-	-	-	-
LEP1GSC059_3600	EQA70859.1	PF07598 domain protein	-	-	+	-	-	-	-	-	-	-	-	-	-	-	-	-	-	-	-	-
LEP1GSC059_4696	EQA70509.1	hypothetical protein	-	-	+	-	-	-	-	-	-	-	-	-	-	-	-	-	-	-	-	-
LEP1GSC194_2880	EQA80457.1	hypothetical protein	-	-	-	+	-	-	-	-	-	-	-	-	-	-	-	-	-	-	-	-
LEP1GSC103_0672	EPG56056.1	PF07598 family protein	-	-	-	-	-	-	+	-	-	-	-	-	-	-	-	-	-	-	-	-

P = Pathogenic species; I = Intermediate species; S = Saprophytic species

**Fig 7:**
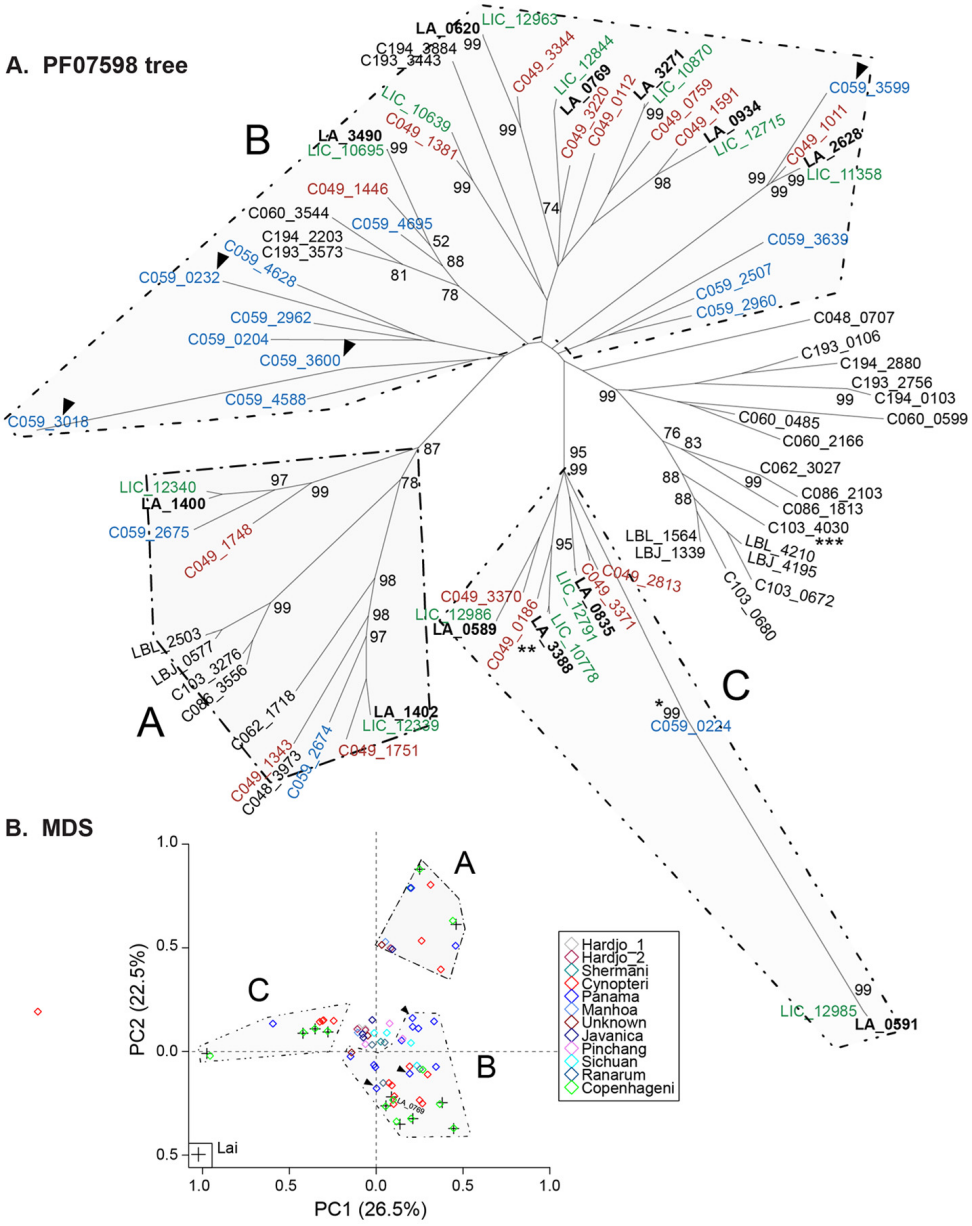
Phylogenetic Relationship of PF07598 Paralogous Family in *Leptospira*. (A) Unrooted bootstrapped phylogenetic tree; (*) Gene duplication event; (**) gene duplication event; (***) gene deletion. (B) Principal components analysis was used to arrange PF07598 family members. Color legend indicates the PF07598 family members from specific serovars depicted as diamonds. Arrowheads indicate *L*. *noguchii*-specific orthologs. Only PF07598 family members longer than 200 amino acids are included in the analysis. Clusters (A, B and C) were defined by K-means clustering with Kendall rank correlation.

To better understand the evolution of this paralogous gene family, a phylogenetic tree of all PF07598 members detected in infectious *Leptospira* was constructed using homologs longer than 200 amino acids shown (Fig 7A). A complex web of lineage specific gene duplications and loss was revealed. For example, as highlighted in (Fig 7A), successive gene duplications and subsequent gene loss have led to four distinct clusters containing proteins in *L*. *interrogans*, *L*. *kirschneri* and *L*. *noguchii* PF07598 family members. The initial duplication event led to divergence of the first orthologous group comprising LEP1GSC059_0224 (*L*. *noguchii*) and LIC_12985 and LA0591 (*L*. *interrogans*) and apparent loss of the corresponding ortholog in *L*. *kirschneri* (*). The second, an *L*. *interrogans*-specific event (**), led to the divergence of two orthologous groups comprised of LA_0589, LIC_12986 and LEP1GSC049_3370, and LA_3388, LIC_10778 and LEP1GSC049_0186, respectively. *L*. *interrogans* Lai 56601 has seemingly lost an ortholog belonging to a group containing LIC10639 and LEP1GSC049_1381 comprised of proteins belonging to *L*. *interrogans* Copenhageni and *L*. *kirschneri*, respectively; and *L*. *kirschneri* 3522CT, *L*. *alexanderi* and both *L*. *borgpetersenii* Hardjo strains seem to have lost an ortholog present in the other pathogenic strains (***). This pattern of species and serovar specific gene duplication and gene deletion occurs throughout the tree. K-means clustering with Kendall rank correlation grouped the *L*. *interrogans* Lai orthologs into three clusters comprising family members with >90% inclusion probability: LA_1400 and LA_1402 (cluster A); LA_0589, LA_0591, LA_0835 and LA_3388 (cluster B); and LA_0620, LA_0769, LA_0934, LA_2628 LA_3271 and LA_3490 (cluster C) (Fig 7B).

#### Motility and chemotaxis

Since motility is required for pathogenesis [150, 151], it is plausible that there are differences in motility and chemotaxis gene content that distinguish infectious from non-infectious species. We identified a total of 76 CDSs encoding proteins involved in leptospiral motility and chemotaxis, using the annotated genome of the *Leptospira interrogans* serovar Copenhageni strain Fiocruz L1-130 as a reference. We established the amino acid sequence identity of CDSs in 20 *Leptospira* genomes based on their respective orthologs in the strain Fiocruz L1-130 genome (S4 Fig; S11 Table). Among these, 37 CDSs were predicted to encode proteins in the basal body assembly and export apparatus; 7 CDSs were predicted to encode proteins in the flagellar hook assembly; 7 CDSs were predicted to encode proteins involved in the filament assembly; and 25 CDSs were predicted to encode proteins in chemotaxis (S4 Fig; S11 Table).

Proteins involved in motility were highly conserved among all the 20 *Leptospira* species according to BLAST analysis and PanOCT ortholog clusters (S4 Fig). The filament is the portion of the flagella which demonstrated the highest amino acid sequence identity, with a mean of 97.9%, 86.4 and 72.4% amino acid sequence identity among pathogenic, intermediate and saprophytic species, respectively (S11 Table). The ORFs that encode the flagellar hook proteins also demonstrate high amino acid sequence conservation, with an average sequence identity of 86.0% and 61.7% in pathogenic and saprophytic species, respectively (S11 Table). FliK, a bi-functional protein involved in determining hook length and modulating export-pathway specificity at the hook–filament checkpoint [152, 153], was the only protein that showed a low level of identity among the three species groups, including within the pathogenic species (69.7%, S4 Fig and S11 Table).

Although CDSs encoding basal body proteins showed the lower amino-acid identity among motility genes, the average identity was high, ranging from 93, 73, and 60% within pathogenic, intermediate and saprophytic species (S4 Fig and S11 Table). In this category, three proteins showed 50% identity or lower when comparing pathogenic species with intermediates and saprophytes. The protein FlgA is involved in the P-ring formation, whereas the FliO and FliJ are involved in the export apparatus. In addition, five CDSs showed an amino acid sequence identity below 50% between pathogenic and saprophytic species, which were CDSs encoding proteins FlgH and FlgL involved in the L- and P-ring formation, respectively, proteins FlgN and FlhX involved in the export apparatus, and the FliG1 protein, which is involved in the motor switch. The P- and L-ring form the outer cylinder and acts as a bushing for the central rod [153, 154] and is believed to participate only passively in the motor mechanism, while the FliG1 protein is believed to be partly responsible for the asymmetrical rotation of the flagella [155].

CDSs encoding chemotaxis proteins are highly conserved among pathogenic species (87% amino acid sequence identity). In contrast, ORFs encoding such proteins are less conserved when comparing pathogenic species with intermediates and saprophytes species groups and have lower amino acid sequence identity (48 and 43%, respectively, S4 Fig and S11 Table). More than 70% of the orthologs of chemotaxis proteins within intermediate and saprophyte species had less than 50% amino acid sequence identity when compared to pathogenic species. Among these proteins, the majority were methyl-accepting protein (MCP) homologs, but include also chemotaxis regulators like *cheA*, *cheR*, *cheB* and *cheY* (S4 Fig and S11 Table). Whereas orthologs associated with construction of the flagellar filament are conserved across pathogenic, intermediate and saprophytic species, ORFs encoding two chemotaxis proteins, one MCP homolog and cheR1, in pathogenic species had no orthologs in intermediate and saprophytic species. Furthermore, one MCP homolog protein in pathogenic and intermediate species had no orthologs in saprophyte species, suggesting a degree of divergence with respect to chemotaxis between pathogenic, intermediate and saprophyte *Leptospira* species.

### Gene regulation and sensory transduction

#### Alternative sigma (σ) factors

σ factors are a class of proteins constituting essential dissociable subunits of prokaryotic RNA polymerase. σ factors provide promoter recognition specificity to the polymerase and contribute to DNA strand separation. All bacterial species have a housekeeping σ-factor (σ^70^) responsible for transcription from the majority of promoters. Most bacteria encode additional alternative σ-factors that redirect RNAP to distinct sets of promoters, which can contribute both directly and indirectly to environmental adaptation and bacterial virulence. In addition to a housekeeping sigma factor σ^70^ (LIC11701, RpoD), all *Leptospira* species have an alternative sigma factors σ^54^ (LIC11545, RpoN) involved in nitrogen and many cellular and environmental regulations, σ^F^ involved in flagella gene expression (LIC11380 (FliA,), 5–11 extracytoplasmic function (ECF) σ factors (σ^E^) involved in regulation of membrane and periplasmic stress, and more than 30 anti-σ regulators (S12 Table).

Leptospiral species differ in σ-factors. First, pathogenic *Leptospira* have two activators (enhancer-binding protein, EBP) for σ^54^, whereas saprophytic *Leptospira* species has only one. σ^54^ is a unique sigma factor that is phylogenetically different from other σ actors. It recognizes a unique −24/−12 promoter sequence (instead of -35/-10 sequence for σ^70^) and its activation always requires an activator, EBP. Signals feed into EBP and activate σ^54^–dependent genes. Each EBP-σ^54^ pairs responds to different signals and activates a set of genes. Our analyses show that while all pathogenic *Leptospira* appear to have two activators (herein named as *L**eptospira* enhancer-binding protein A and B that can be denominated EBP-A and EBP-B, saprophytic *Leptospira* have only one EBP (EBP-A) (S12 Table). Although the upstream signals and downstream targets remain to be elucidated, we speculate that LepA-σ^54^ modulates a group of genes involved in environmental survival for both pathogenic and saprophytic *Leptospira*, whereas LepB-σ^54^ is important for pathogenic *Leptospira* species to adapt to host environment. Second, pathogenic and saprophytic leptospiral species differ in ECF σ factors. Pathogenic and intermediately pathogenic *Leptospira* have 9 to 10 ECFs, and saprophytic species often have 5 ECFs. One ECF (LIC10599) is only found in highly pathogenic *Leptospira*, while 2 ECFs are only associated with saprophytic *Leptospira* (S5 Fig; S12 Table). Lastly, *Leptospira* have more than 30 regulators predicted as anti-σfactors, anti-anti-σfactors, and regulators of anti-anti-σfactors. Although their functions remain unclear, some of these regulators may modulate ECF functions as observed in *B*. *subtilis*. Nevertheless, there are some obvious differences in their distributions among *Leptospira* species (S12 Table). It is conceivable that the ECFs and regulators of σ factors present only in saprophytic *Leptospira* are involved in responding to environmental stress, whereas the ECFs and regulators of σ factors present only in pathogenic *Leptospira* are likely important for *Leptospira*’s life cycle in mammalian hosts.

#### Two Component Systems (TCS)

TCSs are the predominant molecular switches controlling signaling events in bacteria. Typically, TCSs consist of a sensor histidine kinase (HK) and an effector response regulator (RR). A single polypeptide merging both components results in hybrid histidine kinases (HHKs). HKs and RRs are usually found adjacent to each other in the genome. Orphan TCS proteins are unpaired HKs/RRs, which work with their cognate partners that are far apart in the genome. In addition, multistep phosphorelays may include intermediate histidine phosphotransferase proteins (Hpt), adding further complexity to TCS networks. Pathogenic, intermediate and saprophytic *Leptospira* species encode an unusually large and diverse set of TCSs, including orphan HKs and RRs, HHKs and Hpts in addition to classical paired HK/RR systems (Table 8 and S13 Table). Of note, more than 60% of the TCS genes found in *Leptospira* genomes encode non-classical orphan HK, orphan RR, HHK and Hpt proteins (Table 8). Overall, pathogenic species had the lowest average number of TCS genes (76), while saprophytic ones had the largest (102) (Table 8). Genome size-normalized TCS data revealed that pathogenic *Leptospira* species have roughly 35% less TCS genes in comparison to intermediate and saprophytic species (S6 Fig and Table 8). Additionally, pathogenic species had a proportionally lower number of strain-specific TCS genes compared to intermediate and saprophytic species (S7 Fig and S14 Table). We also identified a core set of 16 TCS genes shared among all the *Leptospira* genomes being analyzed (S7 Fig). Half of these TCS genes, conserved among all *Leptospira* species, were orphan HK/RRs (S15 Table). Taking into account their high conservation throughout the species, irrespective of saprophytic or pathogenic mode of lives, this core set of TCSs probably regulates pivotal cellular pathways in *Leptospira*.

**Table 8:** Summary of two component systems identified in 20 *Leptospira* genomes.

Species	Non-Orphan HK	Non-Orphan RR	Orphan HK	Orphan RR	Hybrid HK	Hpt	Total	TCS to genome size ^a^
**Pathogenic**								
*L*. *interrogans*	12	12	13	22	12	3	**74**	15.99
*L*. *kirschneri*	9	9	12	24	12	3	**69**	15.64
*L*. *noguchii*	12	12	12	23	12	3	**74**	15.70
*L*. *alstoni*	15	15	14	24	14	4	**86**	19.38
*L*. *weilli*	13	13	11	19	11	3	**70**	16.41
*L*. *alexanderi*	11	11	12	20	11	3	**68**	16.09
*L*. *borgpetersenii*	12	12	12	21	11	3	**71**	18.22
*L*. *santarosai*	13	13	11	20	11	3	**71**	17.80
*L*. *kmetyi*	18	18	18	25	19	3	**101**	22.85
**Intermediate**								
*L*. *fainei*	19	19	15	27	18	4	**102**	23.79
*L*. *broomii*	21	21	14	26	21	4	**107**	24.34
*L*. *wolffii*	18	18	19	30	16	4	**105**	23.85
*L*. *licerasiae*	15	15	14	36	16	5	**101**	23.98
*L*. *inadai*	17	17	14	28	17	5	**98**	21.98
**Saprophytic**								
*L*. *wolbachii*	17	17	13	33	20	4	**104**	25.47
*L*. *yanagawae*	14	14	16	32	18	4	**98**	24.16
*L*. *biflexa*	14	14	14	34	19	4	**99**	25.05
*L*. *vanthielii*	15	15	12	33	19	4	**98**	23.15
*L*. *terpstrae*	16	16	15	34	22	4	**107**	26.14
*L*. *meyeri*	16	16	13	36	21	5	**107**	25.54

^a^ Number of TCSs / Total genome size (Mb) respective to each *Leptospira* species.

Abbreviations: HK, histidine kinase; RR, response regulator; Hpt, histidine phosphotransferase; TCSs, two component systems; Mb, megabase.

Although there were 15 TCS genes conserved among pathogen and intermediate species, we did not identify genes that were shared between saprophyte:pathogen or saprophyte: intermediate species (S7 Fig). This finding is in agreement with the previous observation that intermediate *Leptospira spp*. are more closely related to pathogens rather than to saprophytes, and that the gene order is more conserved in pathogenic and intermediate species [32]. Among the 15 TCS genes found in both pathogenic and intermediate species, 5 were orphan RRs, 4 were orphan HKs, 2 were HHKs, and only 1 was a classical HK:RR pair (S13 Table).

It is worth noting that *L*. *kmetyi* and *L*. *alstoni* were outliers with respect to being pathogenic species that harbored the largest number of species-specific TCS genes (27 and 17, respectively) and the largest overall number of TCSs within this species group (S7 Fig and S14 Table). In contrast, *L*. *alexanderi* did not contain any species-specific TCS genes (S7 Fig and S14 Table). The seven TCS genes present in all pathogenic species (S16 Table) may represent moieties involved in common signaling pathways and may play a role in host colonization and pathogenetic mechanisms.

## Discussion

Here we used comparative whole genome analysis to answer the overall question, “what makes a bacterial genus pathogenic?” This analysis delineated the definitive phylogenetic relationship among 20 species of *Leptospira*, and demonstrated that infectious species and clades of *Leptospira* contain unique genes that are not found in non-infectious *Leptospira* (summarized in Table 9).

**Table 9:** Summary of Some Key Genomic Differences in *Leptospira* Species that Suggest Role in Evolution from Saprophyte to Infectious Pathogen.

Name of feature	General function (Table/Fig)	Comments
*Rfb* locus	Serovar-specific polysaccharide biosynthesis on lipopolysaccharide (Fig 5; S2 Fig)	*Rfb* loci more complex in pathogenic *Leptospira* than in intermediates and saprophytes, possibly reflecting adaptation to mammalian environments and pathogen-host cell interactions
CRISPR/Cas systems	Defense against exogenous nucleic acids (Table 5; Fig 6)	Found only in pathogenic and intermediate *Leptospira*
Proteolytic enzymes (thermolysin, collagenase)	Degradation of host proteins (eg., complement components, interstitial proteins including collagen) (S9 Table)	Immune evasion, invasion of mammalian host tissues
Sialic acid biosynthetic genes	Predicted to modify proteins with neuraminic acid (Table 3; S3 Fig)	Complete loci found only in pathogenic *Leptospira;* sialic acid-modified proteins predicted to be involved in pathogen-host interactions
Catalase/peroxidase/superoxide dismutase	Detoxification of oxidative radicals (Table 6)	Catalase only found in pathogenic *Leptospira;* superoxide dismutase absent in pathogenic *Leptospira*
Extracellular matrix (ECM)-binding proteins	Adhesion (S8 Table)	Some members found only in pathogenic and intermediates; some found only in pathogenic; some found in all clades. Potentially mediate *Leptospira* adhesion to host cells (in case of infectious *Leptospira*); may be involved movement of *Leptospira* in and through tissue interstitium; may generally promoting invasion/colonization processes
Cobalamin biosynthesis	Production of vitamin B12 (Table 2)	Infectious *Leptospira* predicted to be autotrophic for B12 biosynthesis, predicted to be important during *in vivo* mammalian infection
PF07598 family	Unknown (Table 7)	Upregulated *in vivo;* found only in pathogenic *Leptospira*

In a general sense, the comprehensiveness of this analysis is fundamentally important for understanding large-scale evolutionary mechanisms by which saprophytic bacteria acquire genes to enable infectiousness and pathogenicity. More specifically, considering how complex the genus *Leptospira* is—among the most complex genera of pathogenic bacteria—our analyses indicate that many genetic events over evolutionary time have given rise to pathogenic *Leptospira* of diverse biological properties. The genus *Leptospira* contains non-infectious environmental saprophytes and those members infectious to mammals. Infectious *Leptospira* are subdivided further into phylogenetically separated groups: pathogens (group I) and intermediate pathogens (group II). Previous systems (pathogenomic) approaches began to yield insights into the large scale set of genes that enable infectious *Leptospira* to adhere, invade, colonize, persist, evade the immune system and cause disease in mammalian reservoirs hosts as well as accidental hosts [32, 149, 156]. By identifying novel gene families, differences in pathogen clade-related gene content, and key potential metabolic differences among infectious *Leptospira* species as well as *Leptospira* species that have contrasting potential for causing severe disease, for example, the present work contributes towards the next generation of leptospirosis pathogenesis experimental studies. The data and analyses resulting from this *Leptospira* Genome Project will contribute to new research directions in diagnostics, vaccine and therapeutics development to prevent and ameliorate leptospirosis, with One Health relevance for the health of humans and animals of veterinary importance alike.

This comparative analysis of the genus *Leptospira* assessed phylogenetic relationships among species in several independent ways, including single locus, multilocus [51] and whole genome approaches. All approaches robustly confirmed the separation of the 20 *Leptospira* species into three clades: pathogens, intermediate pathogens and saprophytes; infectious *Leptospira* include members of the pathogen and intermediate pathogen clades. Whole genome analysis produced consistent dendrograms, similar but not identical to those obtained by multi-locus sequence typing (MLST) [51]; MLST has been most useful for characterizing *Leptospira* isolates [22, 51, 70, 157, 158] but also has been used to identify *Leptospira* strains directly in clinical samples. Speciation based on Bayesian analysis of 16s rDNA (*rrs*) gene sequences has become generally accepted [35] for *Leptospira*, especially differentiation of pathogen from non-pathogen (potential contaminant). However, the *Leptospira* 16S rRNA gene is highly conserved so that species cannot be further subdivided. For example, 16S rDNA sequence-deduced phylogeny could not distinguish *L*. *meyeri* from *L*. *yanagawae*. However, the data provided here and elsewhere demonstrate that the gold standard for future definitive taxonomical definition of any *Leptospira* isolate will be based on whole genome sequence-based *in silico* DNA-DNA hybridization [11]. Genome-to-genome distances (GGD) analysis confirmed *in silico* DNA-DNA hybridization results. As previously determined by the classical *in vitro-*performed DNA-DNA hybridization studies, each of the representative strains represents a distinct species (estimated hybridization between pair of strains <70%) (S2 Table). For example, *L*. *interrogans* serovar Icterohaemorrhagiae strain M20 is phylogenetically related to *L*. *kirschneri* strain 3522C (estimated hybridization 42.30% ± 2.53) and *L*. *noguchii* strain CZ214 (estimated hybridization 37.80% ± 2.49), while other pathogenic, intermediate and saprophytic species are distantly related to *L*. *interrogans* (S2 Table). One serovar may belong to more than one species (Fig 5) so that serovar does not have precise taxonomic implications, although the present analysis is limited by not exploring within-species O-antigen loci in the genomic data from the 320 isolates for which genomic sequence information was generated. Finally, serovars belonging to the same species had GGD values higher than 70% DDH similarity. *In silico* DDH values therefore accurately reflect whole genome relatedness and may be used for the purpose of species delineation [159, 160], thus replacing the classical DNA-DNA hybridization technique which, we argue, is now obsolete. The use of genome sequences also provides reusable data and reproducible results. The GC content and a set of core genes (including ribosomal genes) can also be extracted from genome sequences to verify that the data are phylogenetically consistent.

### Lipopolysaccharide *rfb* biosynthetic loci in 20 *Leptospira* species

A remarkable feature of *Leptospira*—one that has often dominated the study of *Leptospira* over the past century—is the serologically-determined variety of *Leptospira* serovars. Serovar identification based on serological agglutination tests previously was the basis of *Leptospira* taxonomy, and continues to be important for understanding leptospirosis epidemiology related to human and animal infection and mammalian reservoir hosts. The present comparative genome analysis of 20 *Leptospira* species, as well as hundreds of additional *Leptospira* whole genome sequences not yet completely analyzed, demonstrates genetic determinants of the antigenic carbohydrates (putatively O-antigens). Therefore molecular analysis of genes in the *rfb* O-antigen locus rather than serological tests will be the future basis of serovar identification.

Leptospiral LPS has long been known to be non-endotoxigenic, the basis for which is thought to be due to unusual modifications of the core Lipid A component of LPS resulting in altered Toll-like receptor-mediated innate immune responses [161–164]. Leptospiral serovar, determined by LPS antigenicity, seems to have some association with mammalian host predilection—for example, serovar Copenhageni with *Rattus* spp., serovar Canicola with dogs [1, 2, 165]—but a causal, mechanistic role here remains to be experimentally demonstrated. Indirect evidence from other microbial systems suggests the speculative hypothesis that the diversity of leptospiral LPS may be driven by environmental ecology-mediated selection pressures [166, 167], such as has been reported in *Salmonella* spp. which has many (>2000) serotypes). The genus *Leptospira* has been reported to contain more than 300 serovars [1, 2, 165], the basis for which remains essentially unexplored.

### Metabolic reconstructions

Here we report the first genomically-predicted metabolic network analysis [168, 169] of *Leptospira*, comparing members of the pathogen, intermediate pathogen and saprophyte clades. These large-scale reconstructions allow classification of the conserved metabolic capabilities (core metabolic network) and the unique metabolic capabilities (pan metabolic network). These reconstructions can be further converted to metabolic models of metabolism to probe metabolic capabilities computationally.

The most striking differences between infectious/pathogenic *Leptospira* and non-pathogens arose in porphyrin and vitamin biosynthetic capabilities. *L*. *interrogans* was shown to have a nearly complete vitamin B12 biosynthetic pathway that enables *de novo* B12 synthesis from an L-glutamate precursor, while *L*. *biflexa* completely lacked this pathway. Only pathogenic *Leptospira*—*L*. *interrogans* and *L*. *kmetyi*—were predicted to have a full folate biosynthetic pathway. These differences in biosynthetic capabilities may allow such pathogens to survive in nutrient-limited niches within the mammalian host. These observations are consistent with previous observations that found that *L*. *interrogans* serovar Canicola can grow *in vitro* in the absence of B12 but not B_1_ [170], but in contrast with observations of others who concluded that pathogenic *Leptospira* could grow in the absence of B_1_ but not B12 [171].

An open question in *Leptospira* biology is why *L*. *interrogans* grows more slowly than do intermediate pathogens and saprophytes, such as *L*. *licerasiae* and *L*. *biflexa*, which grow rapidly in defined EMJH medium [38]. The metabolic network model of *L*. *interrogans* was shown to lack L-glutamate oxidoreductase, an enzyme involved in recruiting ammonia as a nitrogen source [172, 173]), predicting a lower growth yield compared to the other *Leptospira* models in our *in-silico* minimal media analysis. The model of *L*. *biflexa* predicted the greatest yield with this reaction because this *Leptospira* contains the L-aspartate ammonia-lyase reaction allowing it to convert L-aspartate into fumarate and ammonia, in addition to using this component solely for biomass generation. These observations hint at one possible solution to the question of different growth rates, but model-guided experimentation is required to validate this prediction. Predictions made using these metabolic networks depend on an accurate reactome [174], and must be validated experimentally. If the models reported here are further curated and experimentally validated they would be the first such mode for a pathogenic spirochete. Such an approach will yield fundamental insights into diverse metabolic capabilities of this diverse phylum, including *Borrelia* spp., which cause Lyme borreliosis and relapsing fever, and *Treponema* spp., which cause syphilis, yaws, periodontitis and other diseases.

### Vitamin autotrophy

Vitamin B12 (cobalamin) is the largest and most complex of natural organometallic cofactors and coenzymes, its *de novo* synthesis requiring ~30 energetically costly enzymatic steps [175]. Mammals have evolved highly complex, regulated mechanisms to absorb, transport and store cobalamin [176], reminiscent of the baroque processes involved in iron uptake, transport and storage in humans [177] [178]. We found that the genetic machinery for B12 autotrophy is found in infectious but not saprophytic *Leptospira*, leading us to speculate that such autotrophy allows *Leptospira* to infect mammals in the face of B12 sequestration by the host.

All *Leptospira* survive in the external environment, but our analysis predicts that only pathogenic strains make cobalamin *de novo* from L-glutamate, suggesting that this process is critical *in vivo*. Important in this context, cobalamin absorption and utilization in mammals is mediated by an elaborate set of carrier proteins, receptors and transporters [176] that generally are presumed necessary to process and protect this very large molecule. Considering the mechanistic details of cobalamin handling in mammals and the B12 autotrophy of infectious *Leptospira*, we hypothesize that mammalian B12 systems deprive invasive microbes of cobalamin, akin to the role of iron absorption, transport and sequestration known to sequester iron from pathogens, which have evolved siderophore mechanisms to acquire iron *in vivo* in mammalian hosts. Comparative analysis of *de novo* cobalamin biosynthesis in *Leptospira* predicts that infectious *Leptospira* are autotrophic for synthesizing this compound while saprophytes are auxotrophic, and suggests lines of experimentation to explore further the details cobalamin biosynthesis in *Leptospira*. The significance of the absence of complete cobalamin biosynthetic pathways in some group I *Leptospira* remains unclear.

### Detoxification of reactive oxygen species suggests resistance to host defense and differences in ecological niche

Previous comparative biochemical studies of spirochetes demonstrated catalase activity only in pathogenic *Leptospira* (all of which were previously classified inclusively as *L*. *interrogans*), and superoxide dismutase activity only in the saprophytic *L*. *biflexa*; peroxidase activity was present in both clades [179]. An important finding in our comparative genome analysis was that the *Leptospira* catalases, *KatA* and the putative catalase ortholog (LEP1GSC062_4039), were only found in pathogenic *Leptospira*, while the single leptospiral superoxide dismutase gene *sod* was found only in saprophytic *Leptospira*. Catalase has classically been associated with resistance to phagocyte-produced oxidative burst-mediated killing of pathogens (*viz*. hydrogen peroxide), typically intracellularly after phagocytosis. The presence of catalases only in pathogenic *Leptospira* suggests the testable hypothesis that this enzyme class may be involved in intracellular resistance to intracellular host cell killing, following on the published observations of pathogenic *Leptospira* within phagolysosomes [180, 181]; whether pathogenic *Leptospira* survive and proliferate in this subcellular compartment has not been conclusively demonstrated. Conversely, the absence of *sod* in pathogenic *Leptospira* suggests that this clade occupies an environmental niche not exposed to oxidative radicals. Presumably the observation that *sod* is restricted to saprophytes suggests that this clade is exposed to a different context in which oxidative radicals are found in the environment.

### Adhesion

Extracellular matrix (ECM)-binding proteins potentially mediate *Leptospira* adhesion to mammalian host cells, movement of *Leptospira* in and through tissue interstitium, and generally promoting invasion/colonization processes [37, 182–195]. A diverse array of ECM-binding proteins has been identified suggesting a redundancy of adhesion molecules that are probably part of invasion strategies of *Leptospira*. Indeed, many putative adhesins are multifunctional as they bind plasminogen and generate plasmin [185, 196, 197], increasing proteolytic processes associated with infection, or they could participate in immune evasion strategies by interacting with complement regulators (see below). Moreover, several of these proteins are recognized by human leptospirosis serum samples indicating their expression during infection. A caveat is that almost all these putative adhesins have been identified based on binding studies with recombinant proteins. Although site-directed mutagenesis of pathogenic *Leptospira* spp remains difficult, relatively straightforward methods have been developed for functional analysis of putative adhesins genes through gain-of-function studies in *L*. *biflexa* [92] [198][199]. Many putative adhesins are present in saprophytic *Leptospira* but the presence of DNA sequences does not mean that the proteins are expressed. Although non-pathogenic *Leptospira* species may encode the genes for putative adhesins, transcripts of *ompL1* and the protein itself, for example, have not been detected in *L*. *biflexa* serovar Patoc (190). In any event, adhesion to environmental biotic or abiotic structures may be part of the biology of saprophytic *Leptospira* but such a concept remains speculative at this time [200].

Many adhesins are multipurpose proteins as they bind PLG and produce PLA. The generation of fully active PLA, aside from its other functions, also contributes to the enhanced degradation of complement components. Indeed, it has been shown that in *Leptospira*, PLA decreases C3b and human IgG deposition, most probably through their degradation, thereby hampering opsonization, restricting complement antibacterial functions [201]. Another mechanism of complement evasion is through the acquisition of host regulators of complement activation. Surface microbial proteins that bind to complement inhibitors and activate them permit pathogens to inhibit the complement response on the bacteria [202]. Binding of *Leptospira* to factor H (FH), factor H-like protein (FHL-1) and C4 binding protein (C4BP) has been reported [203, 204] and several complement regulators-binding proteins have been identified [205–211]. Lsa23 is an example of multifunctional protein capable of binding ECM, PLG/PLA and complement regulators and should play a role in leptospiral virulence [211].

### Immune evasion via proteolysis of complement

The protein family includes several metalloprotease members that are considered virulence factors in several pathogens [212]. Aureolysin, a zinc-dependent metalloprotease of *S*. *aureus*, acts in synergy with host regulators to inactivate C3 thus potentially inactivating host immune response [213]. Indeed, in the case of *Leptospira*, thermolysins were only found in pathogenic strains. Recently, experimental evidence of the sequence *LIC13322* encoding a putative thermolysin on the direct degradation of complement factors has been reported, suggesting its role in immune evasion by pathogenic leptospiral strains [214]. Thus, it seems that pathogenic *Leptospira*, like other successful pathogens, utilize at least two strategies to circumvent the complement system: acquisition of host complement inhibitors and degradation of complement components, either thorough PLG/PLA generation or by the presence of bacterial proteases.

### Sialic acids

We show here a distinctive presence of the sialic acid cluster in most pathogenic *Leptospira* species and notably absent from intermediately pathogenic and saprophytic *Leptospira* species. This fact suggests a role for sialic acids as virulence determinants. The data shown are consistent with previously published data in which *L*. *interrogans* and *L*. *alexanderi* produced di-acetylated nonulosonic acids and *L*. *santarosai* did not produce identifiable nonulosonic acid species, and this observation could be associated with some species missing particular enzymes of the pathway [215].

The pathogens that contain the whole sialic acid biosynthesis cluster have N-acetylneuraminic acid synthetases predicted to produce legionaminic acids, while other species have a cluster predicted to produce pseudaminic acid. Pseudaminic acid has been shown to be required for flagella biogenesis in *Campylobacter* spp. and *Helicobacter* spp. and function as virulence factors [216]. Leptospiral flagella are located between the inner membrane (IM) and outer membrane (OM) and drive motility [217]. The presence of a pseudaminic acid pathway in all species suggests glycosylation could have a similar role in flagella biogenesis.

Legionaminic acid has been associated with virulence in *Legionella pneumophila* and *Campylobacter coli* where its cell surface location seems to be involved in adhesion, cell-cell interaction and immune evasion [218]. We hypothesize that similar mechanisms occur in pathogenic leptospires.

### PF07598 paralogous gene family

A novel gene family—first identified in a pathogenomic screen of *L*. *interrogans* serovar Lai [219]—was found to have orthologs present in pathogenic *Leptospira* but not in intermediate or saprophytic *Leptospira*. In *L*. *interrogars* serovar Lai, these genes were reported to be upregulated *in vivo* [219] and here we report that the numbers of paralogs varies among the pathogens, with the *L*. *interrogans*, *L*. kirschneri and *L*. *noguchi* having the most. These observations suggest that the PF07598 genes contribute to leptospiral virulence but the mechanism(s) by which they do so remain to be elucidated; no functional annotations for the PF07598 gene family is yet possible. Experimental studies of this gene family will likely provide insight into leptospirosis pathogenesis.

### Motility and chemotaxis

Although motility is essential for pathogenesis of *Leptospira* [150, 151], all *Leptospira* spirochetes, including those belonging to intermediate and saprophytic species, are motile. Consistent with this observation, we found that pathogenic, intermediate and saprophytic species of *Leptospira* have all the genes necessary to assemble a functional flagellar apparatus. Furthermore, the flagella-encoding genes are highly conserved within the genus, indicating that pathogenic and non-pathogenic *Leptospira* do not differ significantly with respect to their flagella apparatus and structure.

In contrast to what we found with regard to motility, there was high diversity with respect to predicted amino acid sequence identity among genes encoding chemotaxis proteins from different species of *Leptospira*. Furthermore, not all chemotaxis proteins are present in all the species, which corresponds to different chemotactic behaviors observed in pathogenic and saprophytic *Leptospira* [220]. The majority of the diversity among the chemotaxis proteins was observed in MCPs, trans-membrane sensor proteins that trigger the intracellular signal transduction in bacterial chemotaxis [221], and located at the cell poles near the basal body and flagellar motor as other chemotaxis proteins [217, 222]. This finding, together with the observation that there is a higher diversity among genes associated with the basal body and that asymmetrical periplasmic flagellar rotation occurs with the interaction of basal body and chemotaxis proteins, suggests that the sensing and chemotactic response regulated by this proteins may impact their survival in specific environments, including their ability to infect a mammalian host.

### Gene regulation

Transcriptional regulation is the most common form of regulation in bacteria, often via a specific transcriptional regulator (activator or repressor) or an alternative sigma factor. In this study, we found that *Leptospira* have only three types of alternative sigma (σ) factors (σ^54^, σ^F^, σ^E^), which is fewer than *E*. *coli* which have genome sizes similar to those of *Leptospira*. Our analysis revealed that there is a distinct difference in the σ^54^ regulatory networks between pathogenic and saprophytic *Leptospira* species. All pathogenic species have two σ^54^ regulatory networks, LepA-σ^54^ and LepB-σ^54^, while saprophytic *Leptospira* only have LepA-σ^54^. Historically, σ^54^ is known to be involved in nitrogen assimilation, and now is well recognized to regulate diverse functions in response to various stimuli [223]. In addition, σ^54^ has also been shown to be essential for infection in some pathogenic bacteria. For example, in another spirochetal pathogen, *B*. *burgdorferi*, the EBP activator, Rrp2, and σ^4^, controls production of RpoS which in turn, governs expression of many virulence factors important for mammalian infection such as OspC [224, 225]. Thus, it is logical to postulate that LepB-σ^54^, is involved in survival in natural environments for both pathogenic and saprophytic *Leptospira*, while LepA-σ^54^ plays a role in survival in the host for pathogenic species. This hypothesis merits experimental testing.

Extracytoplasmic function σ factors (ECF σ factors) are the most diverse alternative σ factors found in many bacteria [226, 227]. Many bacteria contain multiple ECF σ factors. For example, *Pseudomonas aeruginosa* has more than 19 ECF σ factors [226]. Based on sequence analysis, ECF σ factors have been grouped into over 40 classes [226]. Our analyses showed that *Leptospira* have 5–10 ECF σ factors, and pathogenic *Leptospira* have 5 more ECFσ factors than saprophytic species (S11 Table), which is consistent with the more complex life cycle of pathogenic species than of saprophytic species. All *Leptospira* spp. have one copy of ECF31 and ECF43 with unknown functions. Pathogenic *Leptospira* have additional 5 unclassified ECFσ factors. Saprophytic *Leptospira* have one copy of ECF41 and ECF42 that are not found in pathogenic species. Although functions of ECF41 and ECF42-type remain unknown, one report showed that one of the ECF41 σ factors, *SigJ* in *Mycobacterium tuberculosis*, is involved in resistance to hydrogen peroxide [228]. It is unclear whether ECF41 in saprophytic *Leptospira* (LEPBI_I1070) has a similar function as SigJ, and if so, how it contributes to the survival of saprophytic *Leptospira* in the environment.

The activity of ECF σ factors is often regulated by an anti-σ factor, a transmembrane protein that binds and inhibits the activity of ECF σ [229]. Cleavage of anti-σ factor by proteases leads to release and activate σ^E^. Extracellular signals regulate this intramembrane proteolysis often via an anti-anti-σ factor (or called anti-σ antagonist). Our analyses revealed that both pathogenic and saprophytic *Leptospira* have more than 30 σ^E^ regulators. Among them, 17 are only found in pathogenic/intermediate *Leptospira*, while 19 are found solely in saprophytic *Leptospira* (S11 Table). These differences likely reflect the variety of signals sensed by pathogenic and saprophytic *Leptospira*.

In addition to alternative σ factors, both pathogenic and saprophytic *Leptospira* species have many putative transcriptional regulators, far more than is found in other pathogenic spirochetes such as *Borrelia burgdorferi* and *Treponema pallidum* [230, 231]. Our initial analyses of transcriptional regulators among *Leptospira* species did not yield a distinct pattern of correlation with pathogenicity. Further *in silico* and experimental analyses to confirm the prediction and more importantly, to determine their regulatory role in *Leptospira*, is needed.

### Sensory transduction

*Leptospira* species have a high number of two-component sensory systems (TCSs) (70–100) compared to *Borrelia*, *Treponema* and *Bradyspira* (6–20). The number of TCS genes found in a particular *Leptospira* species strongly correlated with the likely diversity of ecological niches that the species encounters, a phenomenon observed in other bacteria [203]. The lower number of TCSs found in pathogenic species may be linked to the process of host adaptation, whereas the larger numbers of unique TCSs in intermediates and, even more in saprophytes, may be instrumental for sensing and adapting to a more diverse range of environmental conditions. Regardless, almost all of the pathogenic species encode more than 70 TCS genes, indicating that the *Leptospira* pathogen requires a highly complex network of signaling processes for its life cycle. Interestingly, the proportion of TCS genes encoding orphan HK/RR and HHK proteins are higher in *Leptospira* (> 60%) than in other bacteria where orphan TCS proteins are unusual. These findings suggest that branched signaling pathways may be relevant in this genus and could confer added physiological advantages to *Leptospira* under specific circumstances.

One limitation in the present analytical approach is the difficulty in defining a robust and confident automatic method to segregate orthologous clusters among all *Leptospira* strains, and especially those that relate to TCS function. As in most *in silico* analyses, further biochemical experiments are needed to confirm the role of the various TCS categories identified in this study. Another limitation of this cross-species comparative analysis is that differences in serovars/strains with *Leptospira* species were not studied; such analyses will be a future priority given the strength of the approach and the depth of existing data. This *in silico* approach will also not identify novel virulence factors nor mechanisms of pathogenesis based on sequence analysis alone.

In summary, the large-scale comparative genomic analysis of 20 *Leptospira* species has provided broad insights into how infectious members of this genus acquired the genes necessary to acquire pathogenicity and virulence, placing these species within a definitive phylogeny. Novel, *Leptospira* species-specific genes and gene families were identified. Genomically-based metabolic reconstruction predictions predict novel adaptation of infectious *Leptospira* to mammals (summarized in Table 9), including sialic acid biosynthesis, pathogen-specific porphyrin metabolism and the first-time demonstration of riboswitch-regulated cobalamin (B12) autotrophy as a bacterial virulence factor. Only pathogenic *Leptospira* contain CRISPR/Cas systems, suggesting not only a potential mechanism for this clade’s refractoriness to gene targeting but also possible novel means to be able to genetically modify pathogenic *Leptospira*. Whether restriction modification systems might contribute to gene targeting has yet to be analyzed in detail, but the publicly available whole genome data sets provided in support of the present work will be contribute to carrying out such analyses. A novel virulence-related genes/gene family epitomized by the PF07598 group of paralogs suggests adaptation and diversification of this protein family within the pathogenic clade. Identifying large scale changes in infectious (pathogenic and intermediate pathogenic) as compared to non-infectious *Leptospira* has yield large-scale, novel insights into the evolution of a bacterial pathogen, provides the basis for new directions in leptospirosis pathogenesis research. It also makes novel genomic and pathogenomic contributions to the field of bacterial pathogenesis, which is of general interest.

## Supporting Information

S1 Fig Pan-genome, core and novel genes of the 20 sequenced *Leptospira* species. The blue and red lines denote the pan-genome and core genes as genomes are added in the order noted on along the x-axis (A). The bars indicate the number of novel gene families discovered for each genome added. The color of the bars illustrate the three main groupings of *Leptospira*: pathogenic (red), intermediate (blue), and saprophytic (green). The number of novel genes discovered with the addition of each new genome (B) was estimated using a pan-genome model based on the original model presented by Tetellin et al. [63]. Purple circles are the median of each distribution (grey circles). Power law (red lines) and exponential (blue lines) regressions were plotted to determine (α), and tg(θ), respectively. The exponent (α) indicates whether the pan-genome is open (α ≤ 1) or closed (α > 1) [83] and tg(θ) denotes the average extrapolated number of strain-specific/novel genes.
(PDF)

S2 Fig Flanking Genes Surrounding the *Leptospira rfb locus* gene clusters. The *rfb* region and flanking CDSs (blue) 9 of pathogenic (A), 5 intermediate (B), and 6 saprophytic (C) representative *Leptospira* species were compared. *rfb* region CDSs are labeled by locus identifier and colored by functional role categories as noted in the boxed key. Gene symbols, when present, are noted above their respective genes. BLASTP matches between CDSs are colored by protein percent identity (see key).
(PDF)

S3 Fig Phylogenetic analysis of leptospiral N-acetylneuraminic (Sialic) Acid Synthetase (NeuB) protein sequences. Maximum-likelihood tree shows pathogens (red lines), intermediates (green lines) and saprophytes (blue lines). Numbers denote node support. A red box highlights those proteins that are part of a complete sialic acid cluster.
(PDF)

S4 Fig Heat map of ORFs encoding 51 motility and 25 chemotaxis proteins identified in analysis of the 20 *Leptospira* genomes. ORFs are identified according to their *L*. *interrogans* serovar Copenhageni strain Fiocruz L1-130 number. The heat map shows the degree of amino acid sequence identity of ORFs with their respective orthologs in the *L*. *interrogans* strain Fiocruz L1-130 genome.
(PDF)

S5 Fig Comparison of ECF Sigma (σ) Factors Among *Leptospira*. Venn diagram showing distribution of ECF σfactors unique or shared among the pathogenic (*L*. *interrogans* L1-130), intermediately pathogenic (*L*. *kmetyi*) and saprophytic (*L*. *biflexa*) species. The number and locus ID of ECF σ factors that are unique or shared among these *Leptospira* species are labeled in each sector of the diagram.
(PDF)

S6 Fig Normalized number of Leptospiral two component systems by genome size. The number of TCS genes was normalized per Mbp genome (y-axis) of representative Leptospiral species (x-axis). See key for shading of pathogenic, intermediate and saprophyte genomes.
(PDF)

S7 Fig Venn diagram showing the distribution of TCS genes among *Leptospira* species. The ratios depicted inside each one of the major groupings, correspond to the number of TCS ortholog genes present in the [majority:all-but-one:all] species of that particular group. True cut-off values for these Figs correspond to the presence of the gene in 50% (majority), 90% (all but one) or 100% (all) of the particular group of species. Sequence clusters that do not match the indicated cut-off value or those from unexpected groupings are included in the “ambiguous grouping” set. Singleton clusters, representing species-specific genes are noted in circles surrounding the Venn diagram.
(PDF)

S1 Table Universal protein markers. (PDF)

S2 Table Estimates of genome relatedness of *Leptospira* species. (XLSX)

S3 Table Metabolites and reactions including exchange and biomass reactions used for metabolic reconstructions. (XLSX)

S4 Table Candidate substrates for the leptospiral TAT protein secretion system. (XLSX)

S5 Table Examination of the -1 position of leptospiral lipobox sequences. (XLSX)

S6 Table Identification of Lipid A biosynthesis proteins in *Leptospira*. (XLSX)

S7 Table A. Identity matrix comparison of lipid A biosynthesis pathway genes across the genus *Leptospira*. B. Homology comparison of amino acid sequences of enzymes involved in the synthesis of cell wall lipid A from *Leptospira* species.
(XLSX)

S8 Table Leptospiral proteins involved in adhesion to extracellular matrix, plasminogen binding and complement evasion. (XLSX)

S9 Table Proteases with a potential role in host-pathogen interactions. (XLSX)

S10 Table A. Amino acid sequence identity comparison of proteins orthologous to *Leptospira interrogans* serovar Copenhageni immunodominant proteins. B. Amino acid identity of leptospiral proteins to Lig proteins and domains.
(XLSX)

S11 Table Comparison of leptospiral proteins involved in motility and chemotaxis. (XLSX)

S12 Table Sigma factors and accessory proteins involved in gene regulation in *Leptospira* species. (XLSX)

S13 Table Inventory of two component systems in *Leptospira* species. (XLSX)

S14 Table Identification of strain-specific two component systems in *Leptospira* species. (XLSX)

S15 Table Core three component system proteins conserved among *Leptospira* species. (XLSX)

S16 Table Two component systems specific to pathogenic *Leptospira* species. (XLSX)
