# Supplementary figures and images for: What Makes a Bacterial Species Pathogenic?:Comparative Genomic Analysis of the Genus Leptospira
Source: PLoS Negl Trop Dis. 2016 Feb 18;10(2):e0004403. doi: 10.1371/journal.pntd.0004403 (PMC4758666; doi:10.1371/journal.pntd.0004403)

# A. Pan-genome Features

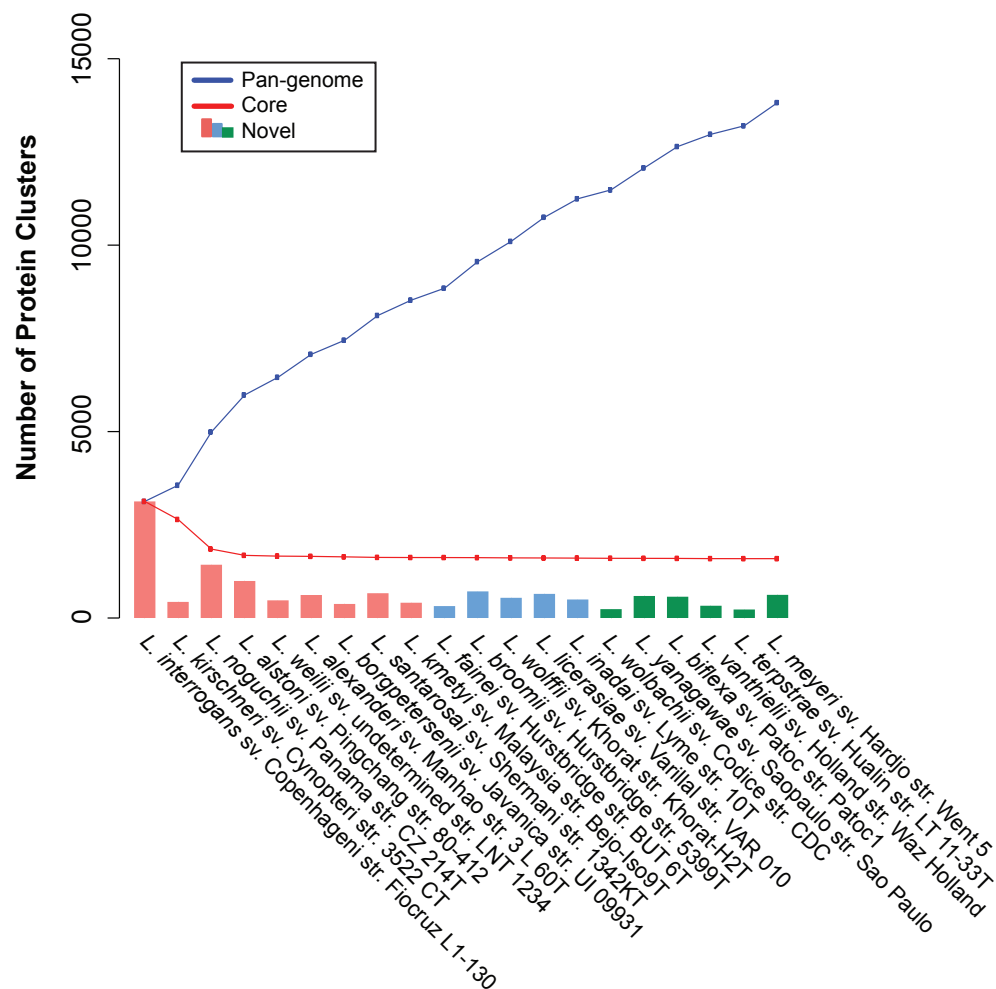

# B. New Genes

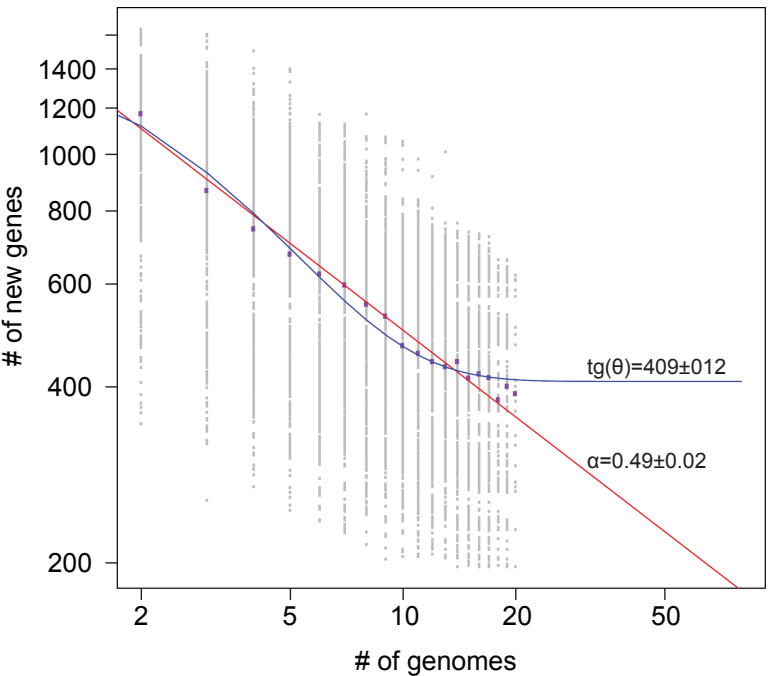

Supplement: S1 Fig — The blue and red lines denote the pan-genome and core genes as genomes are added in the order noted on along the x-axis (A). The bars indicate the number of novel gene families discovered for each genome added. The color of the bars illustrate the three main groupings of Leptospira: pathogenic (red), intermediate (blue), and saprophytic (green). The number of novel genes discovered with the addition of each new genome (B) was estimated using a pan-genome model based on the original model presented by Tetellin et al. [63]. Purple circles are the median of each distribution (grey circles). Power law (red lines) and exponential (blue lines) regressions were plotted to determine (α), and tg(θ), respectively. The exponent (α) indicates whether the pan-genome is open (α ≤ 1) or closed (α > 1) [83] and tg(θ) denotes the average extrapolated number of strain-specific/novel genes. (PDF) [file pntd.0004403.s001.pdf]

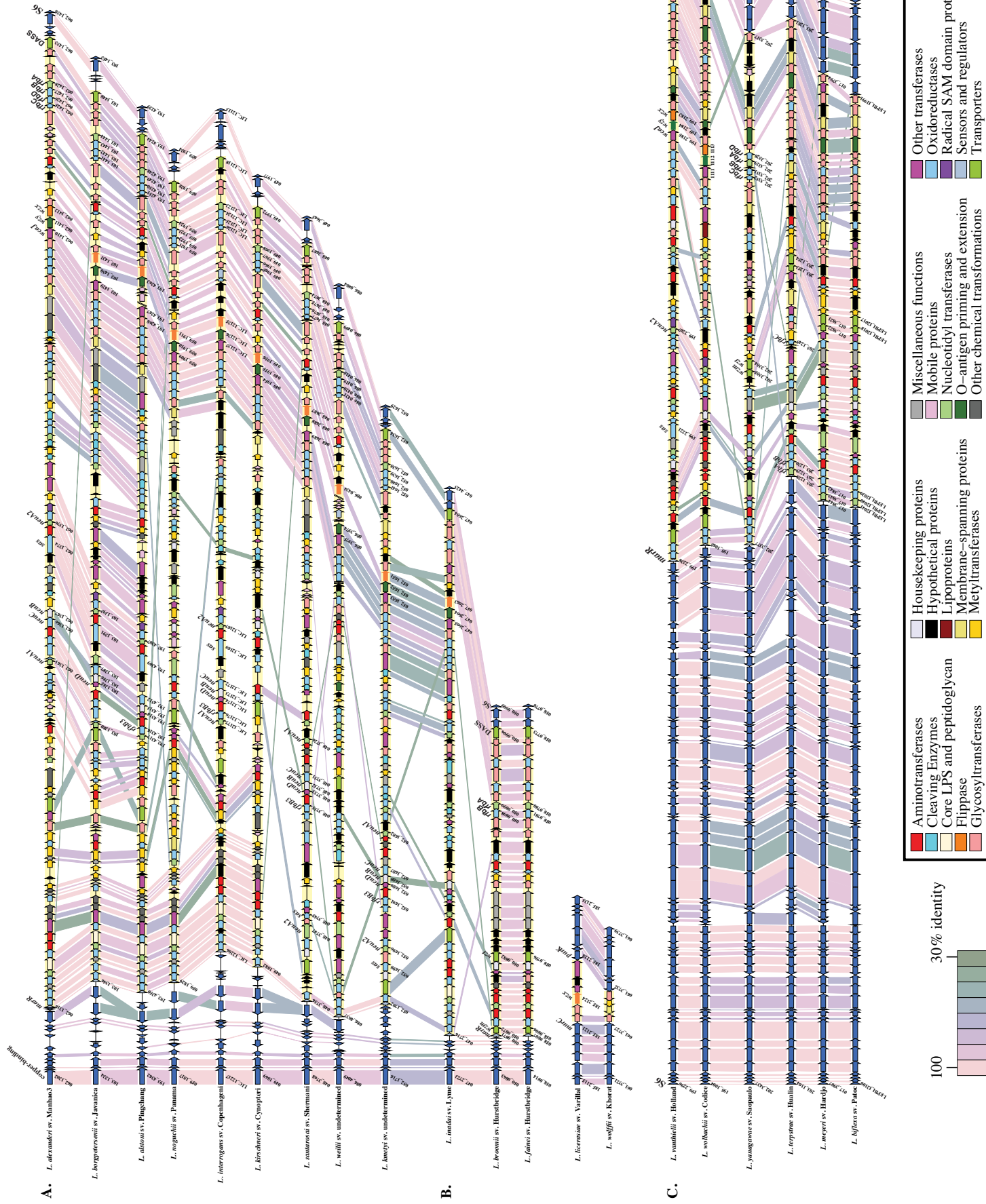

Supplement: S2 Fig — The rfb region and flanking CDSs (blue) 9 of pathogenic (A), 5 intermediate (B), and 6 saprophytic (C) representative Leptospira species were compared. rfb region CDSs are labeled by locus identifier and colored by functional role categories as noted in the boxed key. Gene symbols, when present, are noted above their respective genes. BLASTP matches between CDSs are colored by protein percent identity (see key). (PDF) [file pntd.0004403.s002.pdf]

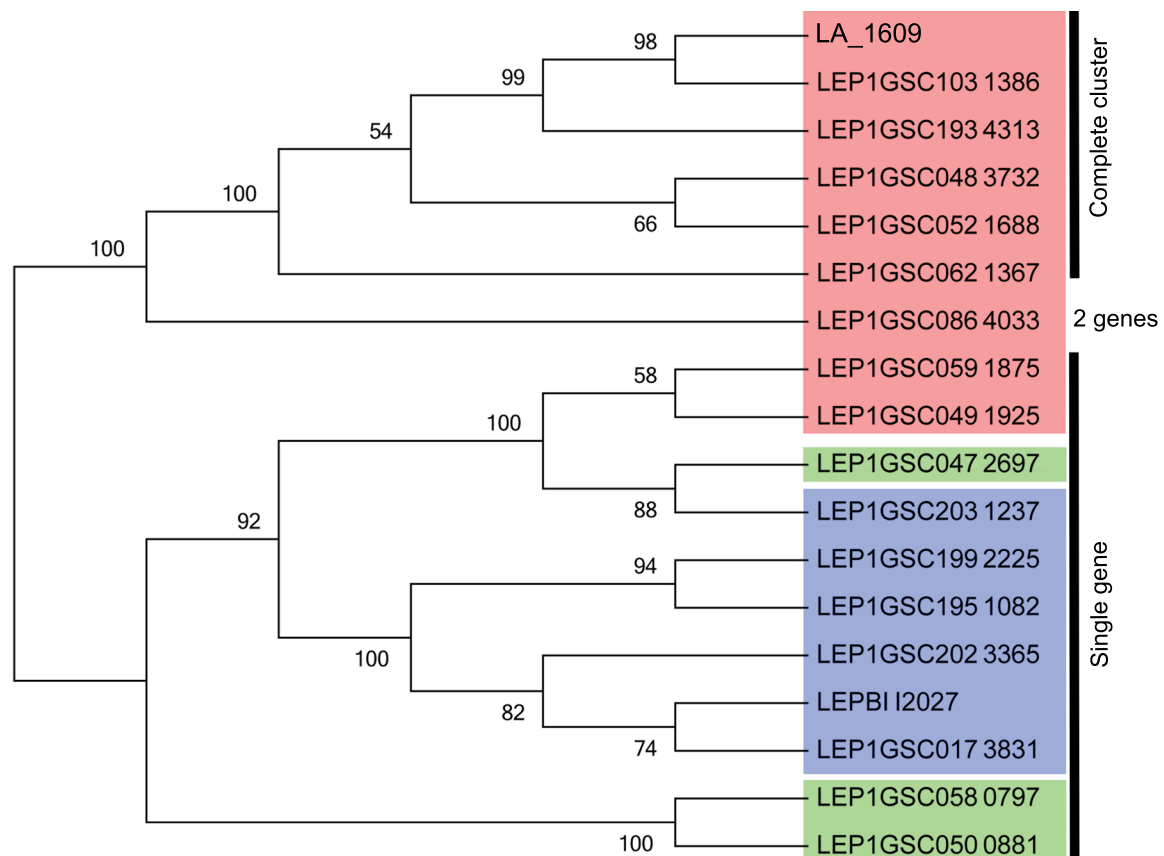

Supplement: S3 Fig — Maximum-likelihood tree shows pathogens (red lines), intermediates (green lines) and saprophytes (blue lines). Numbers denote node support. A red box highlights those proteins that are part of a complete sialic acid cluster. (PDF) [file pntd.0004403.s003.pdf]

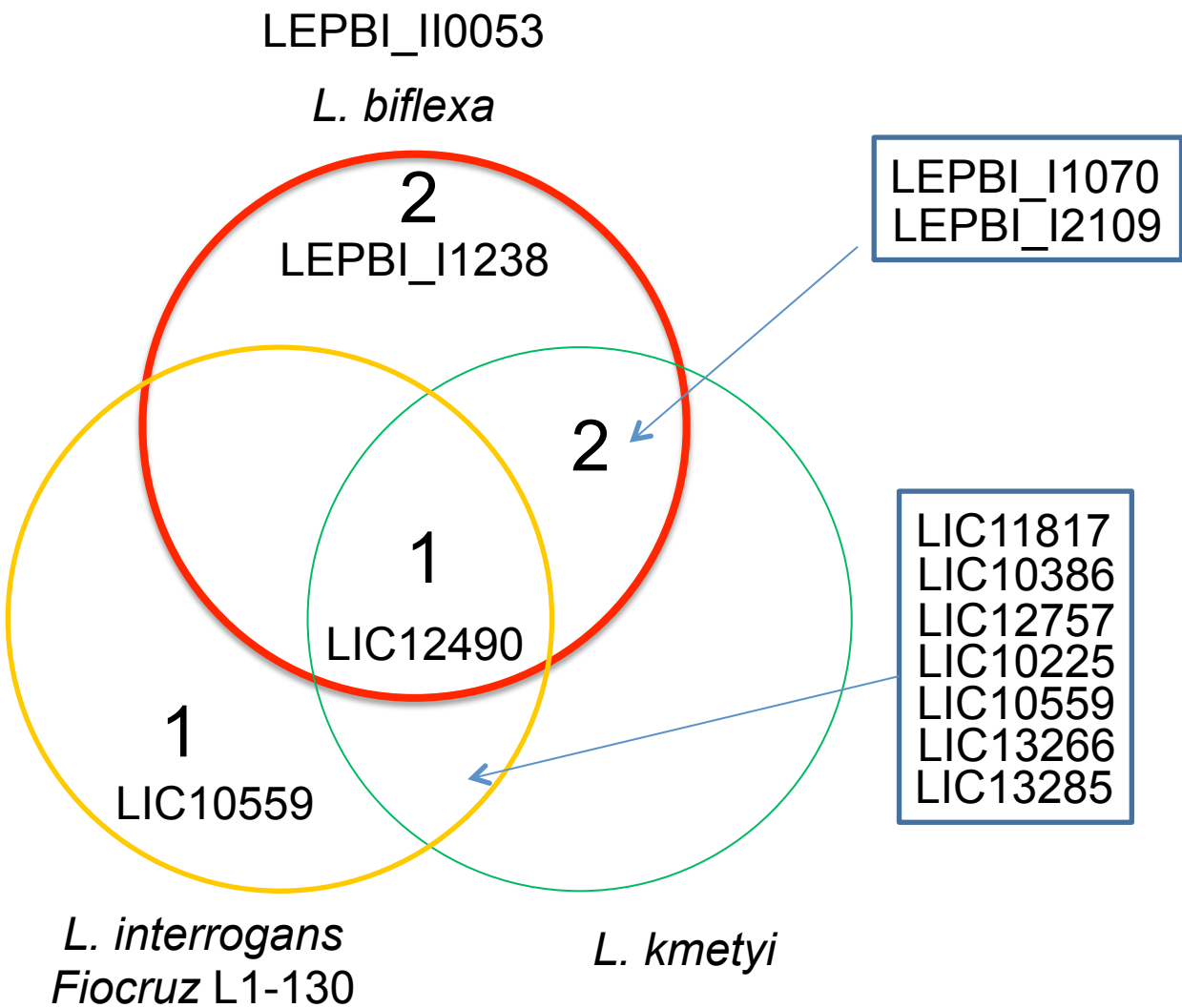

Supplement: S5 Fig — Venn diagram showing distribution of ECF σfactors unique or shared among the pathogenic (L. interrogans L1-130), intermediately pathogenic (L. kmetyi) and saprophytic (L. biflexa) species. The number and locus ID of ECF σ factors that are unique or shared among these Leptospira species are labeled in each sector of the diagram. (PDF) [file pntd.0004403.s005.pdf]

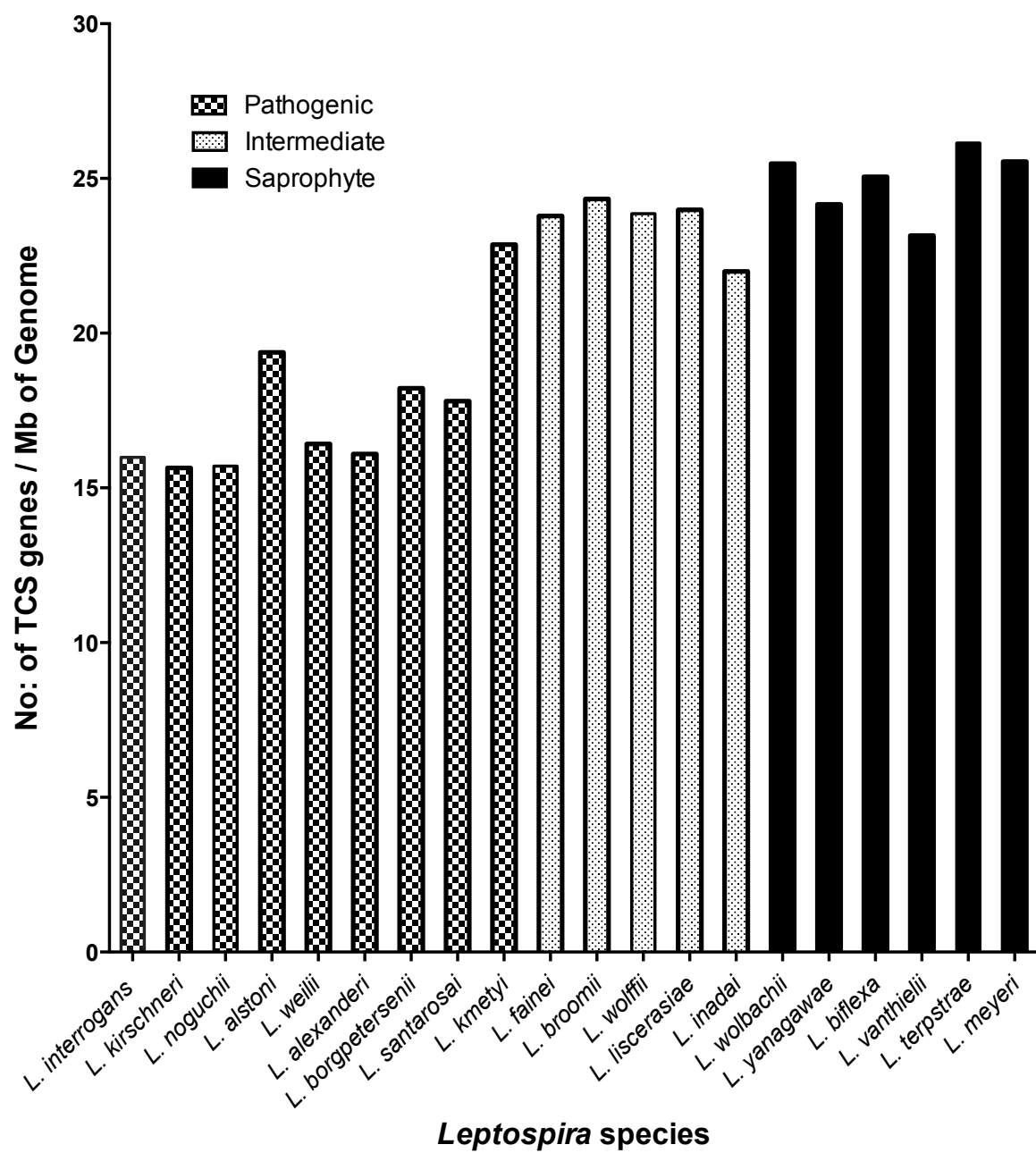

Supplement: S6 Fig — The number of TCS genes was normalized per Mbp genome (y-axis) of representative Leptospiral species (x-axis). See key for shading of pathogenic, intermediate and saprophyte genomes. (PDF) [file pntd.0004403.s006.pdf]

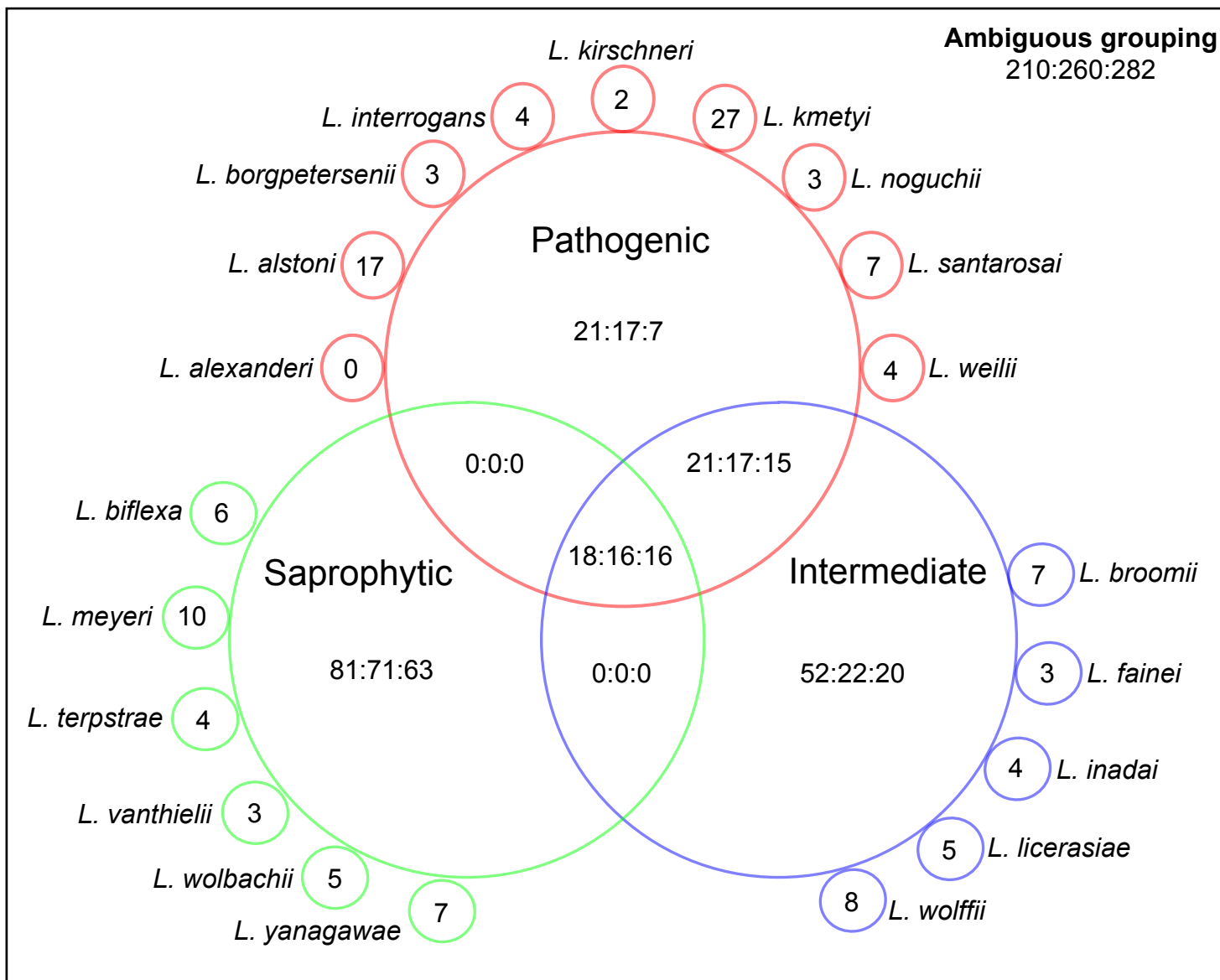

Match definitions: majority:all-but-one:all

Supplement: S7 Fig — The ratios depicted inside each one of the major groupings, correspond to the number of TCS ortholog genes present in the [majority:all-but-one:all] species of that particular group. True cut-off values for these Figs correspond to the presence of the gene in 50% (majority), 90% (all but one) or 100% (all) of the particular group of species. Sequence clusters that do not match the indicated cut-off value or those from unexpected groupings are included in the “ambiguous grouping” set. Singleton clusters, representing species-specific genes are noted in circles surrounding the Venn diagram. (PDF) [file pntd.0004403.s007.pdf]
