# Supplementary material for: What Makes a Bacterial Species Pathogenic?:Comparative Genomic Analysis of the Genus Leptospira
Source: PLoS Negl Trop Dis. 2016 Feb 18;10(2):e0004403. doi: 10.1371/journal.pntd.0004403 (PMC4758666; doi:10.1371/journal.pntd.0004403)
Supplement: S1 Table — (PDF) [file pntd.0004403.s008.pdf]

S1 Table. Universal Protein Markers

Orthologous Group	core HMM accession	gene_sym	Annotation	Reference
COG0173	TIGR00459	aspS	Aspartyl-tRNA synthetase (AspS)	Brown et al., 2001
COG0480	TIGR00484	fusA	Elongation factor G (FusA)	Brown et al., 2001; Santos & Ochman, 2004
COG0187	TIGR01059	gyrB	DNA gyrase B subunit (GyrB)	Brown et al., 2001; Santos & Ochman, 2004
COG0532	TIGR00487	infB	Initiation factor 2 (InfB)	Brown et al., 2001
COG0481	TIGR01393	lepA	GTP-binding protein (LepA)	Brown et al., 2001; Santos & Ochman, 2004
COG0495	TIGR00396	leuS	Leucyl-tRNA synthetase (LeuS)	Brown et al., 2001; Santos & Ochman, 2004; Ciccarelli et al., 2006
COG0504	TIGR00337	pyrG	CTP synthase (PyrG)	Brown et al., 2001; Santos & Ochman, 2004
COG0081	TIGR01169	rplA	Ribosomal protein L1	Ciccarelli et al., 2006
COG0090	TIGR01171	rplB	Ribosomal protein L2 (RplB)	Brown et al., 2001; Santos & Ochman, 2004
COG0087	PF00297	rplC	Ribosomal protein L3	Ciccarelli et al., 2006
COG0094	PF00281	rplE	Ribosomal protein L5	Ciccarelli et al., 2006
COG0097	PF00347	rplF	Ribosomal protein L6P/L9E	Ciccarelli et al., 2006
COG0080	TIGR01632	rplK	Ribosomal protein L11	Ciccarelli et al., 2006
COG0102	TIGR01066	rplM	Ribosomal protein L13	Ciccarelli et al., 2006
COG0093	TIGR01067	rplN	Ribosomal protein L14	Ciccarelli et al., 2006
COG0200	TIGR01071	rplO	Ribosomal protein L15	Ciccarelli et al., 2006
COG0197	TIGR01164	rplP	Ribosomal protein L16/L10E	Ciccarelli et al., 2006
COG0256	TIGR00060	rplR	Ribosomal protein L18	Ciccarelli et al., 2006
COG0091	TIGR01044	rplV	Ribosomal protein L22	Ciccarelli et al., 2006
COG0202	TIGR02027	rpoA	DNA-directed RNA polymerase, alpha subunit (RpoA)	Ciccarelli et al., 2006
COG0085	TIGR02013	rpoB	DNA-directed RNA polymerase, beta subunit (RpoB)	Brown et al., 2001; Santos & Ochman, 2004
COG0052	TIGR01011	rpsB	Ribosomal protein S2	Ciccarelli et al., 2006
COG0092	TIGR01009	rpsC	Ribosomal protein S3	Ciccarelli et al., 2006
COG0522	TIGR01017	rpsD	Ribosomal protein S4 and related proteins	Ciccarelli et al., 2006
COG0098	TIGR01021	rpsE	Ribosomal protein S5 (RpsE)	Brown et al., 2001; Ciccarelli et al., 2006
COG0049	TIGR01029	rpsG	Ribosomal protein S7	Ciccarelli et al., 2006
COG0096	PF00410	rpsH	Ribosomal protein S8 (RpsH)	Brown et al., 2001; Ciccarelli et al., 2006
COG0103	PF00380	rpsI	Ribosomal protein S9	Ciccarelli et al., 2006
COG0100	PF00411	rpsK	Ribosomal protein S11 (RpsK)	Brown et al., 2001; Ciccarelli et al., 2006
COG0048	TIGR00981	rpsL	Ribosomal protein S12	Ciccarelli et al., 2006
COG0099‡	PF00416	rpsM	Ribosomal protein S13	Ciccarelli et al., 2006
COG0184	TIGR00952	rpsO	Ribosomal protein S15P/S13E	Ciccarelli et al., 2006
COG0186	PF00366	rpsQ	Ribosomal protein S17	Ciccarelli et al., 2006
COG0201	TIGR00967	secY	Preprotein translocase subunit SecY	Ciccarelli et al., 2006
COG0172	TIGR00414	serS	Seryl-tRNA synthetase	Ciccarelli et al., 2006
COG0550	TIGR01051	topA	DNA topoisomerase I (TopA)	Brown et al., 2001
COG0533	TIGR03723	tsaD	Metal-dependent proteases with chaperone activity	Ciccarelli et al., 2006
COG0050	TIGR00485	tuf	Elongation factor Tu (Tuf)	Brown et al., 2001
COG0012	TIGR00092	ychF	Predicted GTPase, probable translation factor	Ciccarelli et al., 2006
